# Do seconds make a difference? Investigating strain-specific behavior of yeast in dynamic glucose environments

**DOI:** 10.1186/s12934-026-03029-3

**Published:** 2026-05-13

**Authors:** Luisa Blöbaum, Markus Bünker, Julian Schmitz

**Affiliations:** Multiscale Bioengineering, Bielefeld University, Universitätsstr. 25, 33615 Bielefeld, Germany

**Keywords:** Dynamic microfluidic single-cell cultivation, Strain comparison, Bioprocess development, Saccharomyces cerevisiae, Biosensors, Scale-down, Dynamic environment

## Abstract

**Background:**

When applied for industrial-scale bioproduction, cells are subjected to ever-changing cultivation environments due to bioreactor heterogeneities, which can significantly influence their growth and production behavior. Cultivating and comparing cells and strains under conditions representative of an actual bioprocess instead of laboratory environments is therefore necessary for developing more reliable production strains. Scale-down bioreactors provide an experimental approach for this endeavor, yet they are limited in their temporal resolution and flexibility when it comes to environmental dynamics. Thus, the impact of second-scale differences in dynamic environments on microbial producers remains unexplored. This study uses the advantages of microfluidic single-cell cultivation to compare the growth behavior and intracellular parameters of three yeast strains under dynamic cultivation conditions with alternating phases of glucose excess and limitation at the timescale of seconds aiming to uncover strain-specific performance under as well as adaptation to fluctuating, bioprocess-relevant environments.

**Results:**

Across all three strains, a consistent trend was observed: decreasing glucose availability resulted in reduced growth rates, lower ATP levels, diminished glycolytic flux, and smaller cell sizes. Notably, cells already reached approximately 50% of their maximal growth rate when exposed to growth-promoting glucose conditions for only 10% of the time. Importantly, both growth rate and cell size exhibited an adaptation phase rather than an immediate response to oscillatory glucose supply. Longer exposure to favorable conditions shortened the adaptation phase and resulted in higher adapted growth rates and larger cell sizes.

**Conclusion:**

By leveraging microfluidic single-cell cultivation, this study provides unprecedented temporal resolution of environmental dynamics, enabling direct, strain-specific comparison of cellular responses and adaptation to rapidly changing cultivation environments. Seconds do make a difference. Our findings demonstrate that growth rate is a highly conserved trait under environmental perturbations, which points to an intrinsic robustness of the investigated strains and highlights adaptation as a key determinant of performance in dynamic environments. Together, these insights have important implications for the design of future single-cell experiments and for the development of robust bioprocesses operating under dynamic conditions.

**Supplementary Information:**

The online version contains supplementary material available at 10.1186/s12934-026-03029-3.

## Background

Yeasts play a central role in industrial biotechnology and remain among the most important microbial workhorses in large-scale bioprocesses, where they are used to produce a wide range of commodities, including bulk chemicals, enzymes, and biopharmaceuticals [1]. Despite their long history of industrial application, strain selection remains a critical challenge. Even within a single species, numerous genetically and phenotypically distinct variants and clones may be available, and identifying the strain that performs best under industrially relevant conditions is far from trivial. Performance observed at laboratory scale often fails to translate to large-scale processes [2], underscoring the need for screening strategies that go beyond idealized conditions.

A key limitation of many strain screening approaches is that cells are often characterized under highly controlled, nominally optimal conditions. In contrast, industrial bioprocesses expose microorganisms to a dynamic and heterogeneous environment [3]. Large-scale bioreactors are characterized by gradients in substrate concentration, dissolved gases, pH, and temperature, which arise from differences in the timescales of mixing and metabolic activity [2]. These spatial heterogeneities translate into temporal environmental fluctuations from a cell’s perspective as cells circulate through different reactor zones [4]. As a result, individual cells experience rapidly changing cultivation conditions rather than a steady, well-defined environment.

**Fig. 1:**
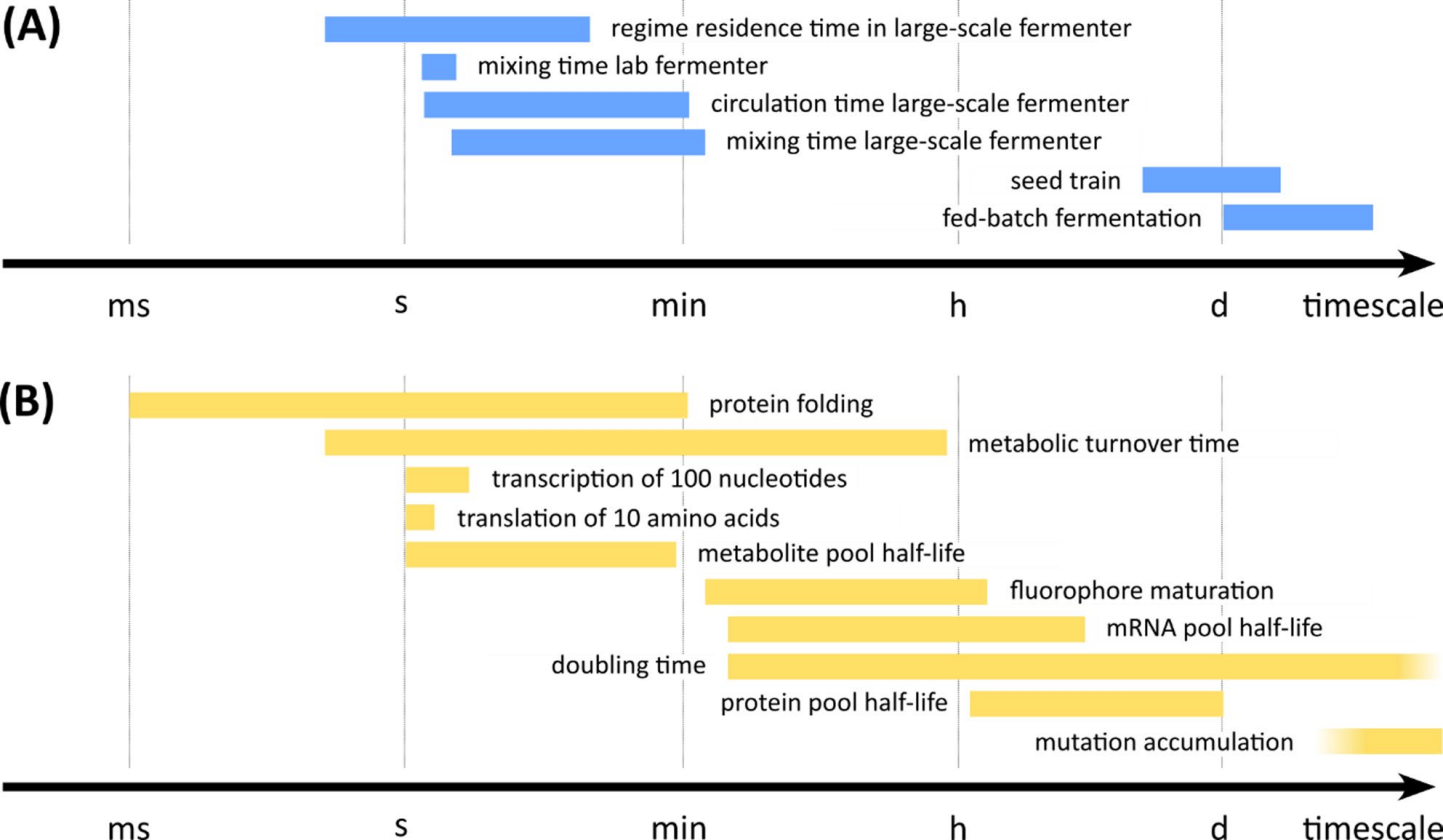
Timescales in microbiology and bioprocessing. **A** Typical timescales in bioprocessing, such as mixing and circulation times in laboratory and large-scale fermenters, regime residence time, seed train preparation, and fed-batch fermentation. **B** Approximate durations of key processes in microbiology, including protein folding, transcription and translation, metabolite and protein pool half-lives, mRNA stability, fluorophore maturation, cell doubling time, metabolic turnover, and mutation accumulation. The horizontal axis indicates timescales ranging from milliseconds (ms) to days (d). For references, see Table S1 in the supplementary information section

Importantly, the characteristic timescales of these spatial heterogeneities as experienced by the cells range from seconds to a few minutes, corresponding to mixing times, circulation times, and residence times in specific regimes (Fig. 1A). These timescales overlap with biologically relevant timescales, such as metabolic turnover, and regulation of transcription and translation [5] (Fig. 1B).

Depending on the relation between the timescales of (repeated) environmental dynamics and microbial response, different reactions in microbial physiology can be expected, as described by Nguyen et al. [6]:

- If microbial response in an observed system is faster than the environmental dynamic, the response reaches a new steady state and shifts between those states alongside the environmental input.
- If they occur on a similar timescale, the response will fluctuate alongside the environment but never reach a steady state.
- If the microbial response is slower than the environmental dynamic, a new physiological steady state will be reached.

However, microbial responses in a fast-reacting system (e.g. metabolic turnover) can propagate towards functions with longer timescales of change (e.g. protein pools), thus influencing cellular physiology, metabolism, and stress responses on the level of seconds to hours. Especially in the context of strain selection and bioprocess development this means that any environmental perturbation regardless of its timescale can lead to long-lasting effects for the cultivated biological system. This was recently demonstrated for *S. cerevisiae* in a study by Torello et al. [7], who analyzed the effect of different timescales of substrate availability dynamics from tens of seconds to nearly hours on three different strains, finding strain-specific differences in the physiological response to the dynamic environment at different timescales. Microbial cells in bioreactors with spatial heterogeneities will experience dynamics at the scale of seconds to a few minutes, that vary by seconds in both duration the symmetry of environmental dynamics. Thus, one question remains to be answered: are differences in short-term dynamics in the order of seconds sufficient to induce measurable and potentially lasting effects on cellular production behavior?

From a bioprocess perspective, this implies that environmental changes occurring on different timescales need to be systematically investigated with respect to their effect on cellular (production) behavior. A clear distinction between the effect of immediate responses and long-term adaptations is required to determine which process dynamics translate into relevant changes in performance under industrial conditions.

In this work, we investigate how small differences in glucose availability dynamics affect microbial behavior. These dynamics occur within a period of 30 s, so on timescales comparable to glucose gradients experienced by cells in bioreactors, and were systematically shifted by 2 s in their symmetry. We employ dynamic microfluidic single-cell cultivation (dMSCC) [8], which enables precise environmental control and rapid oscillations at the timescale of seconds, combined with live-cell imaging at single-cell resolution to capture population-level behavior. Microfluidic systems offer a key experimental advantage in that cellular physiology and environmental conditions are effectively decoupled which clearly benefits the study’s aim to link the effect of environmental dynamics to intracellular physiology. In conventional scale-down systems, cellular metabolism alters the surrounding environment, while the environment simultaneously influences the cells, resulting in a bidirectional coupling between physiology and environmental conditions. Due to the inherent characteristics of dMSCC, individual cells do not significantly alter medium composition, such that environmental concentrations remain effectively independent of cellular metabolism and are experimentally defined and stable regardless of cellular responses [9, 10]. For a more significant readout of cellular behavior dMSCC is integrated with genetically encoded biosensors [11], allowing direct monitoring of the ATP pool and fructose-1,6-bisphosphate (FBP) level, a proxy for glycolytic flux - two key metabolic indicators tightly coupled to glucose availability and cellular wellbeing.

## Methods

### Strain, media, and cultivation conditions

The ethanol producing *S. cerevisiae* strains CEN.PK113-7D, Ethanol Red, and PE2 were cultivated in Verduyn medium (“Delft Medium”) [12] with pH adjusted to 5 with KOH. All three strains were equipped with genome-integrated fluorescent biosensors monitoring the level of fructose-1,6-bisphosphate as proxy for glycolytic flux (GlyRNA) and ATP-level (QUEEN-2 m) [11]. For pre-culture performed in shake flasks the medium contained 20 g/L glucose, while for microfluidic experiments the glucose concentration was set to 50 g/L for excess conditions and 10 mg/L for limitation conditions, similar to previously published data [7]. Each pre-culture was started by inoculating 10 mL of pre-culture medium with scrape-off from frozen cryo culture. Shake flask cultivation was performed in 100 mL baffled shake flask at 30 °C and 120 rpm for approx. 24 h. On the next day the pre-culture was diluted to an OD_600_ of 0.2 to 0.3 with pre-culture medium for microfluidic cultivation. Cryo stocks were prepared by inoculating a 100 mL baffled shake flask of Verduyn medium (10 mL working volume) from agar plate and cultivating up to an OD_600_ of 2 (still exponential phase) at 30 °C and 120 rpm. Cell suspension was mixed with glycerol to a final concentration of 20% glycerol (v/v), shock-frozen in liquid nitrogen and stored at −80 °C.

## Microfluidic device fabrication

The microfluidic cultivation device was fabricated in-house from a silicon wafer mold [13]. Polydimethylsiloxane (PDMS) base and curing agent were mixed in a 10:1 ratio, poured onto the wafer, degassed until no bubbles were visible anymore and baked for 2 h at 80 °C. Afterwards the chip was cut from the wafer and trimmed to the correct size. Inlets and outlets were introduced manually via biopsy puncher and the PDMS mold as well as a glass slide were rinsed with isopropanol. By use of oxygen plasma both PDMS mold and glass slide were surface-activated and bonded. For a more detailed description of each step, advice, and optimization see [14].

### Chip characterization

**Fig. 2:**
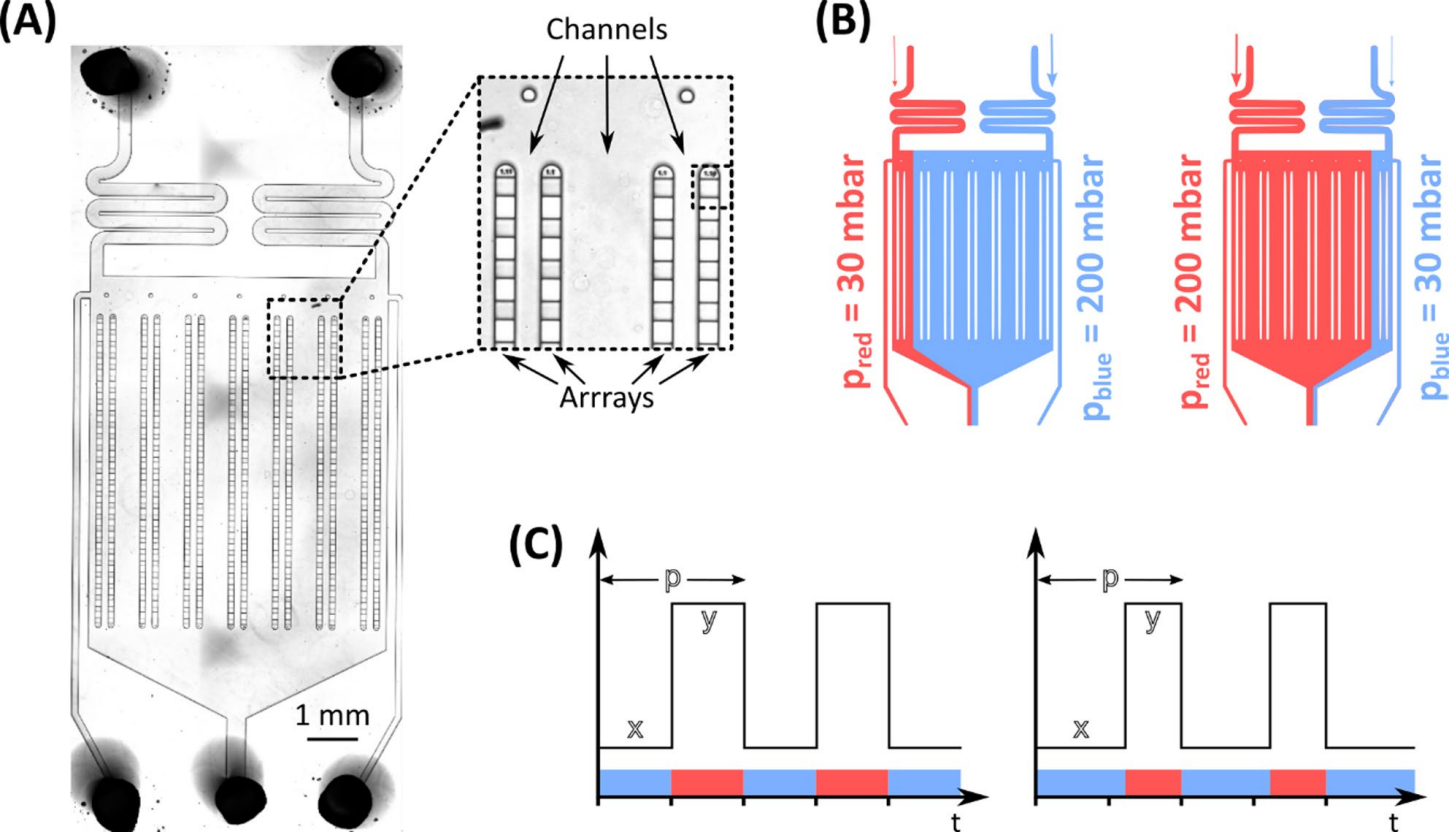
Operating principle of the dMSCC chip. **A** The microfluidic cultivation device consists of 7 parallel running cultivation arrays, each holding 27 monolayer growth chambers with a cultivation area of 80 × 90 μm² and a height of 4 μm. **B** To establish a stable laminar flow profile on-chip the inlet pressure of the cultivation media is altered between 30 mbar and 200 mbar depending on whether limitation or excess is desired. **C** Dynamic cultivation conditions with a fixed 30 s oscillation period (p). The durations of stress (x) and optimal (y) phases are varied within each 30 s cycle between experiments, enabling comparison of strain-specific responses and the impact of subtle timing differences in environmental dynamics

The microfluidic cultivation device holds seven pairs of cultivation arrays with approx. 30 monolayer growth chambers (80 × 90 × 4 μm³) per array (Fig. 2A). Entering from the inlets, two different cultivation media can be introduced to the chip. The inlet pressure settings translate into a certain flow profile on-chip: Equal pressure positions the laminar flow boundary in the middle of the chip while unequal pressure shifts the laminar flow boundary towards the lower pressure inlet (Fig. 2B). The outermost arrays on the left and right sides are exposed to unchanged cultivation conditions throughout the entire experiment and therefore serve as the positive and negative controls. For stable oscillation between the two cultivation conditions the inlet pressure is periodically changed between 30 mbar and 200 mbar. To analyze whether the environmental changes reach the cultivation chambers or only the supply channels, oscillations are tested with yellow-fluorescent dye. 500 mg/L of the dye are solved in 70% ethanol (v/v) oscillated against non-colored ethanol with a frequency of 5 and 15 s (see [14] for further experimental details). For chip mapping the fluorescence value of different cultivation chambers with specific positions (Fig. 2A) was monitored over multiple cycles.

### Dynamic microfluidic single-cell cultivation

The microfluidic cultivation device is mounted on an automated microscope (Nikon Eclipse Ti2, Nikon) and inoculated manually. The cultivation temperature is regulated to a constant 30 °C by a microscope incubation cage (OKO-H201, OKO Lab). For cell loading the chip is flushed with both cultivation media via the inlets and cell suspension is introduced via the outlet. For a detailed protocol of the inoculation procedure see [13]. After an initial growth phase of 4 h under optimal cultivation conditions (except for the negative control) regular oscillations are initiated. With a fixed oscillation period of 30 s the cells are exposed to varying durations of glucose limitation and excess e.g., 27 s of limitation and 3 s of excess (Fig. 2C). In this context, “time spent in excess/limitation” refers to the duration a cell experiences excess or limitation conditions within the constant 30 s oscillation period. Likewise, when comparing different ratios of excess and limitation within the fix 30 s period the term “longer/shorter excess/limitation duration” is used. The duration of limitation and excess is stepwise adjusted by 2 s between consecutive experiments.

For the subsequent investigation of growth and biosensor answer at least five cultivation chambers per condition are analyzed via live cell imaging using 100x oil objective (CFI P-Apo DM Lambda, Nikon). Phase contrast images were captured every 8 min for 100 ms and an intensity of 15% using the microscope’s DIA illumination. Fluorescence images were captured every 32 min with the acquisition settings in Table 1 using a LED-based light source (Sola SE II Set, Lumencor):

**Table 1:** Fluorescent capture details for all fluorescent proteins used in this study. ^a^ required for GlyRNA, ^b^ required for QUEEN-2 m

Protein/Channel	Excitation wavelength (nm)	DM (nm)	Emission wavelength (nm)	Exposure (ms)	Intensity (%)	Gain (-)
mCherry^a^/RFP	462/40	593	640/75	60	10	1.5
cpGFP^b^/GFP	472/30	495	520/35	400	15	1.0
mTurquoise2^a^/CFP	420/40	455	470 Longpass	80	10	1.0
cpGFP^b^/uvGFP	390/40	425	520/35	800	25	1.0

^a^ required for GlyRNA, ^b^ required for QUEEN-2 m

### Image analysis

The image data was first processed and analyzed using a previously published pipeline [13] in FIJI [15]. At first, images of one position were pre-processed by stabilizing, tilting and cutting. The background was subtracted for better fluorescence quantification using a rolling ball algorithm. Individual yeast cells were segmented using a StarDist 2D model [16] with a self-trained model. For each detected cell, size, shape descriptors and gray value of each fluorescent channel were recorded for further data analysis. The scripts for image analysis and the model are available via GitHub (https://github.com/lucatorep/Robustness_Microfluidics).

### Data analysis

As a first step, single-cell data of all chambers in the same condition were combined into one file for further analysis.

### Growth rate

Growth rate was determined using a linear regression fit to the logarithmic curve of the total cell area. The specific maximal growth rate (µ_max_) within a 3 h window was determined by shifting the calculation window frame by frame after the onset of the dynamics. The initial growth rate (µ_ini_) was determined in the time frame of 1 h after the onset of the dynamics. For strain comparison, the value was normalized with the maximum growth rate of the excess control condition of the same experiment.

### Adaptation time

The adaptation time was calculated as the point of intersection between the linear fits for the growth rate 1 h (µ_ini_) after onset of the dynamics and the specific maximal growth rate (µ_max_).

### Biosensor ratios

Intracellular dynamics of ATP-level and glycolytic flux were followed using fluorescent biosensors. The ratio of the uvGFP to the GFP signal of the biosensor QUEEN-2 m was computed to determine the ATP-level. As a first step, the biosensor readout of GlyRNA was determined for each cell, by computing the ratio of CFP to RFP. To describe the distribution of the biosensor signals within the population (see Fig. 4B and C), the first step was to calculate the median signal and the interquartile range of 25–75% of all observed cells for one condition at one timepoint. In a second step, the mean of those medians was determined in the fixed time window of 10–18 h of cultivation (6 h after onset of environmental dynamics), resulting one plotted data point. For strain comparison, the values in the cultivation time between 10 and 18 h were normalized with the value of the excess control condition of the same experiment within the same timeframe.

### Cell size

Similar to the biosensor ratios, the median cell size (Fig. 4D) and the interquartile range of 25–75% of all observed cells for one condition were determined for one timepoint and then averaged using the mean in the fixed time window of 10 to 18 h of cultivation (6 h after onset of environmental dynamics) to describe the cell size distribution. For strain comparison, the values in the cultivation time between 10 and 18 h were normalized with the value of the excess control condition of the same experiment within the same timeframe.

## Results

### Mapping spatial pulse variability in microfluidic growth chambers

Observing effects of environmental dynamics at the timescale of seconds requires a decoupling of physiology and environmental conditions as well as a precise and validated manipulation of cultivation conditions. The constant exchange of cultivation medium within the microfluidic device guarantees for the necessary decoupling. Therefore, we characterized the excess-limitation-dynamics in the applied microfluidic cultivation device with respect to phase duration, resolution and relative amplitude of the pulses inside the growth chambers. By mapping various chamber positions on-chip, a comprehensive overview of the real timescale of mass transfer characteristics was obtained which guaranteed meaningful experimentation over the course of the subsequent study.

**Fig. 3:**
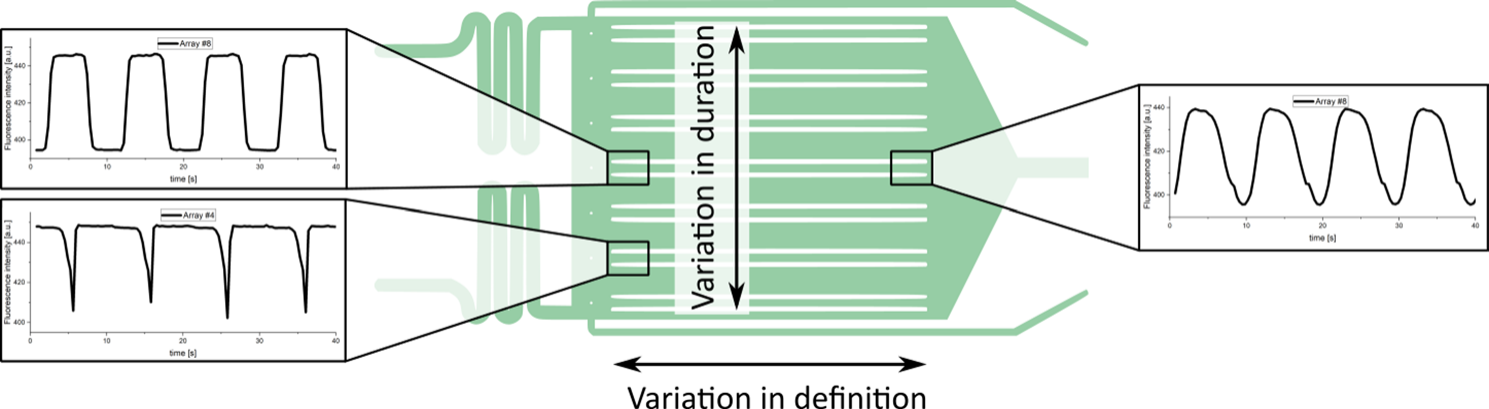
Variation in oscillation duration and definition across the microfluidic chip. The schematic illustrates a microfluidic cultivation chip with parallel arrays. Environmental profiles differ in two dimensions: Duration, varying vertically across arrays (left panels), and definition, decreasing horizontally along the chip length (top panels)

Figure 3 shows how the symmetric oscillation with a phase duration of 5 s propagates differently along both the width and the length of the chip. Inspection of the upper sections of the parallel arrays reveals that the intended 5-s dynamics occurs exclusively at the center of the chip. Shifting the laminar boundary layer requires a few seconds, depending on flow and shifting distance, so that cultivation chambers within the peripheral arrays experience a prolongation or shortening of the respective pulse duration. Higher inlet flows will minimize this temporal delay, with the trade-off being higher medium consumption and decreased cell retention. Along with the longitudinal axis of the arrays, the concentration profile loses definition. This phenomenon results from the propagation of the profile through the chip. By increasing the flow rate these effects can be reduced yet maintaining definition along the full array length would demand excessively high flow rates.

As the following experiments required very defined dynamics, only the first 10 cultivation chambers of each array were used. Furthermore, the chip characterization revealed a shift of 2 s between the middle array pair and the immediate neighboring pair on each side at the chosen flow settings (see Fig. S1 for more details). Those arrays were used for analysis, leveraging the shift in time for experimental parallelization.

### Changes in the timescale of seconds influence performance

**Fig. 4:**
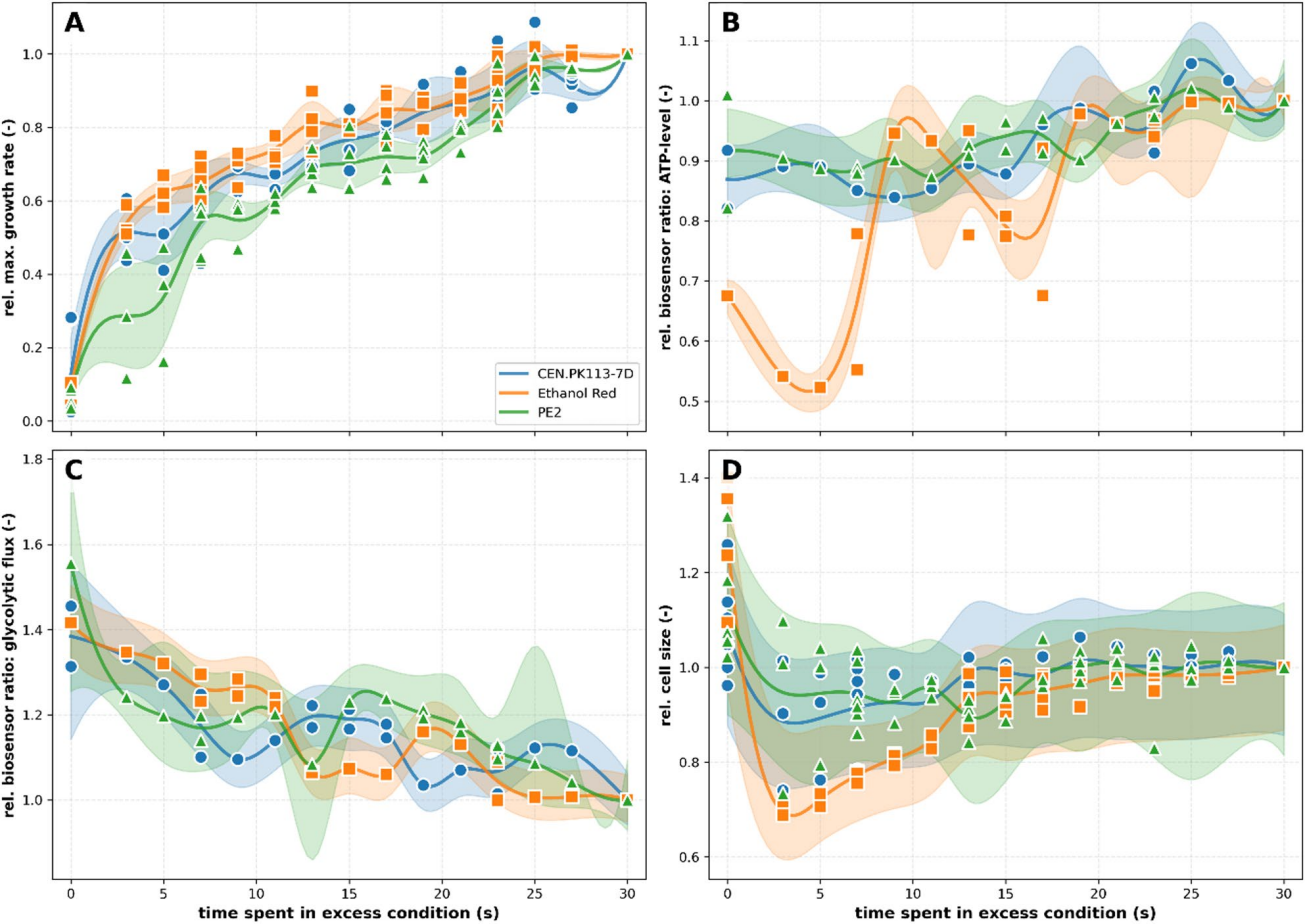
Population-averaged cellular performance of the three examined yeast strains for different times spent in excess condition during the timeframe between 10 and 18 h of cultivation. **A** Relative µ_max_ calculated on population level. **B** Relative ratio of QUEEN-2 m measuring the ATP-level. **C** Relative ratio of GlyRNA measuring the FBP level as indicator for glycolytic flux. **D** Relative cell size. For details on data analysis, see the according methods section. All data were normalized to the respective excess condition. In case of two or more experiments for one dynamic condition, the spline curve was drawn using the mean of the data points or the averaged IQR, respectively

This section compares the performance of the three yeast strains with respect to the growth rate, the cell size, the glycolytic flux and the intracellular ATP level under the influence of dynamics in glucose availability. Glucose concentrations of 50 g/L for excess condition and 10 mg/L as limiting condition were chosen according to a previous study [7]. These concentrations were further selected to reflect process conditions rather than to deliberately target the onset of specific physiological regimes. In industrial bioprocesses, cells are likely to experience glucose concentrations in the range of 50 g/L, which motivated the choice of this level. As this concentration is significantly higher than the Monod constant (Kₛ), it can be considered representative of clear excess conditions. As 50 g/L > > K_s_ for glucose in yeast [17], excess response can be assumed. Likewise, in yeast bioprocesses glucose is typically depleted over time, making starvation a realistic representation of a second physiological regime. However, since the aim of this study was to enable biosensor expression, a minimum level of energy supply was required. The previous study by *Torello et al.* has demonstrated that cells are still capable of expressing biosensors at glucose concentrations as low as 10 mg/L [7]. Yet, 10 mg/L of glucose are less than K_s_ but still enabled growth (Fig. 4A), placing it in the limitation regime. Time spent in excess and limitation condition varied by 2 s within a 30 s period. Data on biosensors and cell size were obtained after 10 h of cultivation to prevent any technical bias due to a limited cell number in the first half of the experiment. All data were normalized to the value of the excess control condition for better comparability between strains.

The strains have different maximal growth rates (µ_max_) (Fig. S2A) but show a similar trend when normalized (Fig. 4A). Strain-independently, a clear decline in µ_max_ with less time spent in glucose excess is observable. Yet, with only 3 s access to glucose (10% of each oscillation period) all strains still preserve approx. 50% of µ_max_.

A clear tendency in the population-averaged intracellular ATP level can be observed as well: Longer glucose excess duration results in higher ATP levels (Fig. 4B). For PE2 and CEN-PK113-7D the overall increase of ATP level between constant glucose limitation and excess is as low as 10%, since already during limitation 90% of the max. ATP level according to the biosensor was maintained. Ethanol Red is an exception as the overall intracellular ATP level is lower than for the other two strains (Fig. S2B). Moreover, Ethanol Red exhibits only 70% of the max. ATP level at constant limitation and even ATP levels < 60% for limitation phases of 23 s to 27 s. Additionally, there are differences in the ATP level distribution within the populations of the three strains: Ethanol Red has two distinct subpopulations, one with higher and one with lower ATP levels, while the other two strains show no signs of macroheterogeneity. Ethanol Red’s subpopulations are present from the very start of the experiment (Fig. S11), and under both constant and dynamic glucose conditions (Fig. S12 and S16). Cells from one subpopulation are keeping their level of ATP over generations. Therefore, the ratio of one subpopulation to the other at the start of the experiments influences the population-averaged ATP level shown in Fig. 4B.

The biosensor GlyRNA was used to monitor the level of fructose-1,6-bisphosphate (FBP) as a proxy for glycolytic flux. Here, a lower ratio indicates a higher glycolytic flux [11]. Again, a uniform tendency for all strains with just minor irregularities can be observed (Fig. 4C). The longer the excess phase, the higher appears to be the glycolytic flux. The increase of roughly 40% in biosensor readout between constant limitation to excess is identical for all strains. Differences in the distribution of GlyRNA-sensor readout, however, exist between the strains: Ethanol Red and CEN.PK113-7D are homogeneous in distribution, while PE2 has a cell that shows high sensor readouts, occasionally forming distinct subpopulations (Fig. S13 and S17).

To conclude Fig. 4D shows the relation between glucose availability and cell size. CEN-PK113-7D and PE2 exhibit changes in cell size only when glucose excess duration is < 17 s, showing a gradual reduction to approximately 90% of their original cell size. In contrast, Ethanol Red again displays a distinct response pattern similar to the ATP dynamics. With decreasing time in glucose excess down to 15 s, cell size gradually declines by about 10%. When glucose excess duration is less than 15 s, a much more pronounced reduction occurs, with cell size decreasing to roughly 70% of the regular size. However, contrary to ATP levels, the cell size is not showing any subpopulation for Ethanol Red, with the exception of a shoulder towards smaller cells resulting from budding events (Fig. S14 and S16). PE2 shows a similar shoulder in the cell area distribution (Fig. S17), which is more widespread in conditions with short durations in glucose excess conditions. This heterogeneity can explain the differences between the experiments for PE2 under conditions with short durations in glucose excess (Fig. 4A and D). Under conditions of constant glucose limitation, all strains respond uniformly by increasing their cell size.

### Cellular adaptation to environmental dynamics

Growth rate and cell size are the product of fundamental cellular processes that operate on different timescales ranging from seconds over minutes to hours. Therefore, changes in cell size are unlikely to reflect immediate cellular responses but rather indicate physiological adaptive processes unfolding over time. Likewise, adaptation in growth rate takes place on the scale of minutes to hours, however, under suboptimal conditions growth might stop immediately. Based on this hypothesis, we systematically analyzed growth rate and cell size dynamics throughout the cultivation, encompassing the phase prior to the onset of dynamics, the initial dynamic phase, and the continued dynamic regime until the end of the experiment. This time-resolved analysis allows adaptation dynamics to be distinguished from short-term regulatory responses. Yet it is important to keep in mind that although environmental perturbations take place on the second-scale, the resolution of the analyzed physiological outcomes range from minutes to hours, as microscopic images were taken only every 8 min during dMSCC cultivation.

**Fig. 5:**
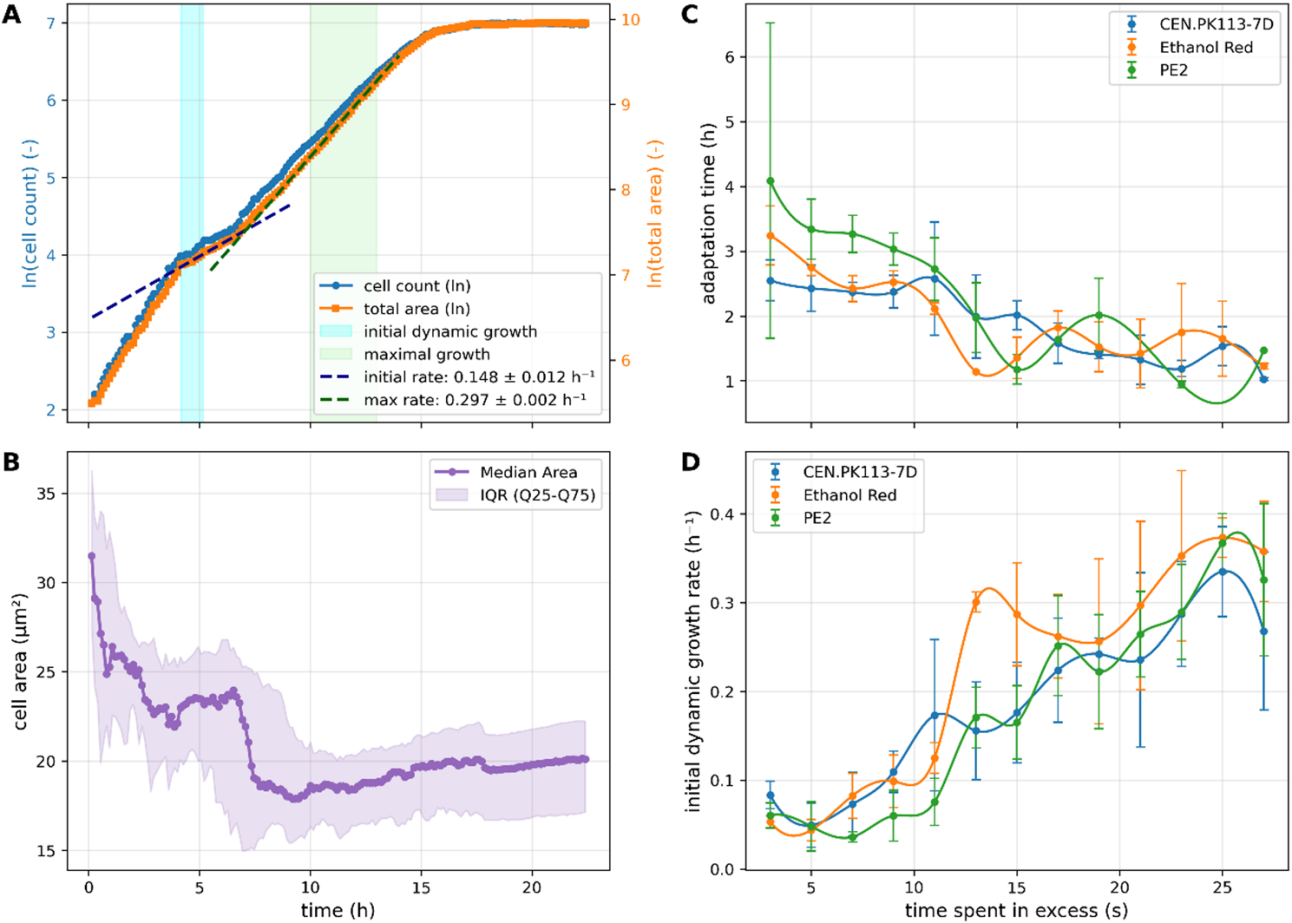
Duration and growth rate in the adaptation phase. **A** Growth curve of Ethanol Red with 9 s spent in excess (9 s/21 s oscillation) based on cell count and total colony area. The initial growth rate (µ_ini_) was calculated over the first hour after the onset of the dynamics at 4 h cultivation time (blue), while the timeframe for µ_max_ is marked in green. **B** Cell size development of cultivation in A. **C** Duration of the adaptation phase against the time spent in excess condition within a constant 30 s oscillation period. Data are shown for any experiment, in which µ_ini_ < µ_max_ and µ_ini_/µ_max_ < 0.9. **D** Mean of µ_ini_ of all strains and conditions. Error bars indicate standard deviation. Data of A and B calculated for the same condition for the other two strains can be found in Fig. S9

Upon the onset of dynamics, a drop in growth rate was generally observed in all conditions. Yet some microcolonies, especially those with less time spent in excess conditions, exhibited a more differentiated reaction in growth rate upon onset of dynamics: as exemplary depicted in Fig. 5A, the growth rate decreases and stays on a lower level for 3.1 h before increasing again by 100% without reaching the pre-dynamics-level. A similar pattern is observed for cell size dynamics. At the beginning of the cultivation, cell size remains at baseline levels (Fig. 5B). With the onset of dynamics, cell size stays largely constant throughout the adaptation phase. A pronounced decrease in cell size occurs only once the growth rate begins to increase again and approaches the adapted growth rate (µ_max_), indicating that changes in cell size are closely linked to the completion of the adaptation process rather than to the initial oscillatory stimulus.

To evaluate the influence of the different limitation-excess cycles, the duration of the observed adaptation phase and the initial µ (µ_ini_) within the first hour upon the onset of dynamics were therefore quantified for each of the three yeast strains. Again, a consistent tendency can be observed: Longer excess durations lead to shorter adaptation phases (Fig. 5B). Likewise, the growth rate during adaptation phase increases with longer time spent in excess conditions until growth in the adaptation phase is indecipherable from adapted growth (Fig. 5C). Comparing the three strains shows that PE2 has the longest adaptation phase when exposed to long limitation phases while CEN-PK113-7D and Ethanol Red behave very similarly.

Adaptation in µ and cell size is observed across all strains (Fig. S3-S8), but no consistent behavior among the strains could be identified in glycolytic flux or ATP level. For example, Ethanol Red is the only strain that has a pronounced reaction in glycolytic flux in shorter times spent in excess: In the first hours in dynamics, the GlyRNA biosensor indicates an increase in activity, reaching a steady level only after 10 h of cultivation (6 h after start in dynamics) (Fig. S6). Furthermore, only for CEN-PK113-7D adaptation can be observed in the ATP-level: The QUEEN-2 m-signal decreases slightly for 1 h after onset of dynamics in conditions with very short time in excess (Fig. S3).

## Discussion

### Chip characterization enables fast dynamics and parallelized experiments

Precise characterization of the applied microfluidic device enabled multiple oscillations to be performed within a single experiment. For instance, the reproducible 2 s temporal shift between neighboring arrays was strategically leveraged to increase the parallelization of experiments, maximizing data yield and enabling a more detailed assessment of cellular responses to dynamic conditions.

Compared to earlier chip designs and characterizations, our approach offers several key improvements. Previous studies [8] typically implemented a single oscillation profile with a maximal temporal resolution of 5 s. In those studies, measurements from central arrays were averaged without regard to position, masking spatial differences within the device. In contrast, our current characterization enabled multiple oscillation profiles on the same chip with a higher temporal resolution of 3 s, facilitated by higher chambers (4 μm vs. 0.8 μm) that allowed for faster medium exchange by convective flow rather than solely diffusion.

Still, the definition of the imposed pulse and its intended duration gradually diminish along the length of the arrays (Fig. 3). This is mainly an effect of the volume exchange within the chambers: The first chambers are exchanged very fast, as only fresh medium is convectively transported in laminar flow and the diffusion gradient is maximal. However, the content of the first chamber is transported with the laminar flow, passing through the following chamber and decreasing the diffusion gradient. A laminar layer between the chambers and the channel emerges along the array, transporting the former chamber contents. This results in smoothed oscillation profiles rather than sharply defined transitions like they occur at the top of the chip (Fig. S1), which has to be considered in the experimental design.

Consequently, the selection of the analyzed cultivation chamber has a substantial impact on the experimental outcome. Particularly for short limitation intervals, glucose concentrations in chambers towards the end of the array may not fall below levels required for sustained growth, thereby effectively reducing the severity of limitation. When investigating a defined oscillation profile, the first chambers on the central arrays provide the highest spatial and temporal fidelity of the intended flow profile. As distinct oscillations were essential for this study, only the first ten chambers of each array were analyzed to minimize this potential source of bias. Nevertheless, a more detailed correlation between spatial position and biological response will be required in future studies to fully exclude positional effects within these chambers.

Interestingly, the smoothed profiles observed toward the end of the chip may not necessarily represent a disadvantage. Cells in these regions are exposed to less abrupt environmental transitions, which could result in more moderate physiological responses. Moreover, large-scale bioreactor gradients are typically not sharply separated but rather blend into one another. In this context, the smoothed environmental profiles at the distal end of the chip may even resemble large-scale conditions more closely. However, this hypothesis requires careful validation through detailed chip characterization and direct comparison with gradients observed in industrial bioreactors.

### Second-scale glucose dynamics shape microbial performance

Comparing the strains, Ethanol Red had the highest absolute µ_max_ when faced with dynamics, followed by CEN.PK113-7D and PE2. This growth ranking is in accordance with previous studies [7]. When grown in dynamic glucose environments, all strains exhibited a decreased growth rate with decreasing time spent in excess condition within the 30 s oscillation. Thus, changing environmental glucose dynamics by merely 2 s influences cellular processes on other timescales. Notably, all strains grew faster than would be expected by the time-weighted average of their constant-condition growth rates (excess vs. limitation). This was also previously observed for periods on the timescale of tens of seconds for yeast in glucose dynamics [7] as well as *Escherichia coli* [18] and *Corynebacterium glutamicum* [8] in nutrient dynamics.

Faster growth rates as an effect of longer excess durations could be expected, yet this relationship is not linear (Fig. S10). While the relation between glucose concentration and growth rate is usually described by the Monod kinetics, applying this model to the determined growth curves results in a poor correlation (R^2^_CEN.PK113−7D_ = 0.8396, R^2^_Ethanol Red_ = 0.9045, R^2^_PE2_ = 0.8434) (Fig. S10). One important assumption in Monod kinetics is the existence of a single limiting substrate, in this case glucose. Since for the microfluidic experiment the same medium is applied as for the flask experiments as well as constantly perfused through the device, every other component of the medium is expected to be present in a non-limiting concentration. Hence, deviation from Monod kinetics must originate not only from glucose concentration, temporal availability and transport but also from intracellular storage capacity like glycogen or trehalose [19, 20] and increased maintenance [21]. Given that these aspects likewise influence the growth in repeated environmental fluctuations, we conclude that Monod does not describe the relationship between time spent in high glucose concentrations and growth rate properly. Furthermore, Monod also does not account for adaptation processes that might enhance growth under very limiting conditions.

Less time spent in glucose excess generally led to slightly lower average ATP levels in all strains (Fig. 4). Strikingly, CEN.PK113-7D and PE2 kept their ATP level at approx. 90% sensor readout of the positive control level, despite growing in severe glucose limitation. Ethanol Red on the other hand, showed two distinct subpopulations with different levels of ATP, which were not connected to the tested condition. While initial reactions (timescale seconds to minutes) in ATP levels to changing glucose situations were reported in literature [13, 21, 22], *S. cerevisiae* is also described to have a very stable ATP level across cell cycle and different carbon sources [23, 24]. Under nutrient depletion, especially trehalose synthesis functions as a buffer as it restores phosphate availability by liberating inorganic phosphate [25, 26]. As one of the central metabolites in energy metabolism, a reliable homeostasis seems to be a plausible trait of a robust strain. Yet, a stable pool size does not necessarily imply unchanged ATP consumption behavior, as CEN.PK113-7D in dynamic environments was reported to have increased ATP expenditure to maintain growth [27] or possibly stress response and futile cycling [28]. As the setup of this work tracks the ATP pool size, only the net of production and consumption but not the underlying metabolic fluxes and the ATP turnover can be assessed. Consequently, ATP consumption and production rates may change while ATP homeostasis is still given. All in all, CEN-PK113-7D and PE2 show a better ability to maintain high ATP levels in increasingly glucose-limiting conditions than Ethanol Red does (Fig. 4B).

The application of the GlyRNA sensor enabled investigation of the FBP pool, the size of which strongly correlates with the glycolytic flux [29]. As the duration of glucose-excess conditions decreased, all strains exhibited an approximately 40% reduction in population-averaged glycolytic flux according to the sensor readout. As can be expected, flux under glucose excess is higher than under limitation. PE2 occasionally shows population heterogeneity in the sensor readout, yet no clear connection to the tested conditions can be made. Adaptation processes in glycolytic flux were described before: Glucose limiting conditions lead to a higher content of high affinity transporters, while low affinity transporters are degraded [30]. Under glucose fluctuation conditions, however, *S. cerevisiae* was found to downregulate upper glycolysis enzymes and glucose transporters, while upregulating enzymes of the lower glycolysis [28]. Likewise, trehalose futile cycling under repleting conditions assures balanced glycolytic flux between ATP consuming upper-part and ATP producing lower-part [31, 32]. The observed decline in glycolytic flux in our data correlates strongly with the duration of glucose excess, suggesting that in fluctuation-experiencing cells, glucose availability plays a major role in glycolytic flux.

The observed alterations of cell size depend on respective time spent in excess conditions hint at three different physiological states. During constant limitation all three strains increase their cell size which contrasts classic studies reporting reduced cell protein/size with slower growth [33]. Yet the observed trends hint at an initiating transition towards a quiescent state. Normally, cell cycle transition and therefore growth is delayed depending on the cell’s size while simultaneously size is a result of environmental conditions that can be monitored by the cell through certain mechanisms [33]. Here, it appears that at harsh limiting conditions (10 mg/L) cell cycle progression and size increase are differently affected. This leads to some kind of transitional state where increase in cell size seems to be less diminished in comparison to deceleration of cell cycle progression, leading to bigger cells that are still able to divide, yet at a much slower rate. The second physiological state can be observed under predominantly limiting dynamics conditions. Here, cells show a decreased size, which however increases with more time spent in excess. This underlines an enhanced nutrient proficiency of yeast cells under limiting conditions again based on change in glycolysis and the expression of high-affinity transporters which resembles the data found concerning the glycolytic flux [27]. Lastly, cells experiencing predominant excess conditions resume to normal cell size in relation to permanent glucose excess environment.

Subpopulations were observed for Ethanol Red (ATP level) and PE2 (glycolytic flux sensor, cell size), while those functions in CEN.PK113-7D were more homogeneously distributed in comparison. For ATP levels and glycolytic flux sensor readout, the heterogeneity was already visible at the start of the microfluidic experiment. Even though the resolution of those subpopulations was limited at the start of the experiments, due to the low number of starting cells, the subpopulations are an indicator that the population heterogeneity emerged already in the preculture and is thus inherent to the tested strain and not the tested dynamic glucose condition.

Overall, we see differences in performance when looking at the absolute values for growth, ATP level, glycolytic flux and cell size (Fig. S2). Yet, these differences are rather strain dependent and do not result from environmental dynamics because the general behavior under dynamic conditions is the same (see normalized data in Fig. 4). All strains were able to maintain growth, ATP level and glycolytic flux well when faced with glucose dynamics. This physiological feature likely contributes to their continuous application by the scientific and bioproduction community.

### Dynamic glucose exposure reshapes growth and cell size within hours

All three strains adapt their growth rate to dynamic environments within 2 to 3.5 h (Fig. 5A and D, Fig S9), allowing them to achieve steady growth with 50% of excess µ_max_ when only 10% (3 s) of the time is spent in beneficial glucose conditions.

Minden et al. [27] showed a reduced growth rate and activated general stress response upon first exposure to fluctuations for CEN.PK113-7D, while fluctuation-adapted cells had higher internal growth capacities at the cost of increased maintenance. While that study applied different timescales of dynamics (minutes), the underlying mechanism of initial response and physiological adaptation are likely similar. Minden et al. [27] also reported a response in gene expression for 3 h after a single dynamic in non-adapted cells. With a duration of 2 to 3.5 h, the adaptation duration of growth rate matches this timescale, indicating that the observed reaction is likely due to the remodeling of the proteome. To further support this hypothesis, another study showed that in glucose dynamics, the proteome of yeast is changed towards a reduced amount of enzymes of the glucose uptake and upper glycolysis [28].

The duration of adaptation in growth rate and cell size depends on the respective duration of the limitation and excess phases within the dynamic conditions. More time in limiting conditions led to a decrease in µ_ini_ and a longer adaptation duration (see Fig. 5). This response seems reasonable as sustained exposure to favorable glucose conditions is expected to lessen the need for extensive proteomic remodeling as well as providing more energy for adaptation and needing less maintenance to encounter the effects of dynamics. This effect could also translate into the limitation phase, as longer periods for generating reserve carbohydrates like glycogen under excess conditions might allow for carbon and energy maintenance when nutrients are depleted [34].

Just like growth rate also cell size adapts to a certain extent to the dynamic conditions. The observable decrease in size does not take place over the whole adaptation process that is detectable for the cells’ respective growth rate but rather happens in a short period of time at the end of this adaptation phase right before µ_max_ is reached. All three strains show a decrease in their size when less time spent in excess conditions which is an already well described adaptation mechanism in yeast cells [35]. Interestingly, cell size is closely determined by glucose metabolism not vice versa. Consequently, adaptation of size has to take place after other processes already have been adapted, which is also reflected in our data. Clear differences in the occurrence of adaptation in cell size between the strains can be seen. Ethanol Red shows the most distinct adaptation towards less favorable glucose availability by decreasing its cell size to a minimum of 70% in the least favorable glucose conditions.

Comparing the adaptive behavior of all three investigated strains, Ethanol Red has the most dynamic response in its functions upon onset of oscillating environmental conditions. This can especially be seen in the decrease in cell size (see Fig. 5) where Ethanol Red has the biggest amplitude in its immediate cell size decrease (compare Fig. S4 & S8) as well as the shortest response time. When looking at the long-term adaptation towards shorter time spent in excess (Fig. 4 & S2), Ethanol Red shows the biggest relative and absolute variability in its cell size. Already in previous studies Ethanol Red was observed to be very robust in its growth rate and production behavior when faced dynamic glucose perturbations [7, 36]. This pronounce and fast adjustment in intracellular parameters can be understood as a mechanism of Ethanol Red’s robustness [37, 38].

## Conclusion

The comparison of the three yeasts trains CEN.PK113-7D, PE2, and Ethanol Red showed distinct differences in their absolute performance while the overall trend was identical: The longer the cells were exposed to growth-promoting glucose conditions, the higher the growth rate, the higher the ATP level and the higher the glycolytic flux. Concerning growth rate and cell size, all strains showed clearly noticeable adaptation whereupon the time it took for the cells to adapt to the environmental dynamics increased with shorter glucose excess phases. Overall, growth rate transpired as highly conserved when it comes to environmental perturbations, as already with minimal glucose exposure half maximal growth rates were achieved.

By applying microfluidic single-cell cultivation, this study achieves unprecedented temporal resolution of dynamic cultivation environments, enabling direct comparison of strain-specific responses and adaptations. This approach not only revealed that seconds do make a difference but also identified a distinct two-phase adaptation process with associated growth rates. While previous studies were largely restricted to pulse-response experiments or environmental changes occurring over tens of seconds to minutes they fail to capture biologically relevant second-scale perturbations. However, due to larger volumes these approaches often enable more extensive omics-analyses. Our findings demonstrate that such short timescales are critical, highlighting the complementary potential of combining classical scale-down reactors with microfluidic systems to exploit the strengths of both approaches for the development of novel bioprocesses.

## Supplementary Information

Supplementary Material 1.

## Data Availability

The datasets used and/or analyzed during the current study are available from the corresponding author on reasonable request.

## Abbreviations

ATP: Adenosine tri phosphate
dMSCC: Dynamic microfluidic single-cell cultivation
DM: Dichroic mirror
FBP: Fructose-1, 6-bisphosphate
PDMS: Polydimethylsiloxane
µ_max_: Maximal specific growth rate
µ_ini_: Initial specific growth rate after onset of dynamics
K_s_: Substrate affinity constant
