# Supplementary material for: Do seconds make a difference? Investigating strain-specific behavior of yeast in dynamic glucose environments
Source: Microb Cell Fact. 2026 May 13;25:125. doi: 10.1186/s12934-026-03029-3 (PMC13179603; doi:10.1186/s12934-026-03029-3)
Supplement: Supplementary file 1 — Supplementary Material 1. [file 12934_2026_3029_MOESM1_ESM.docx]

# Supplementary information

# Do seconds make a difference? Investigating strain-specific behavior of yeast in dynamic glucose environments

Luisa Blöbaum^1^, Markus Bünker^1^, Julian Schmitz^1,#^

^1^Multiscale Bioengineering, Bielefeld University, Universitätsstr. 25, 33615 Bielefeld, Germany

The supplementary information include:

- Table S1: Reference table for timescales in microbiology and bioprocessing.
- Figure S1: Characterization of the applied microfluidic chip concerning flow profile definition.
- Figure S2: Cellular performance of the three examined yeast strains in different times spent in excess condition.
- Figure S3: Growth, cell size and ATP level of the strain CEN.PK113-7D at 9 s spent in excess conditions over the course of cultivation.
- Figure S4: Growth, cell size and glycolytic flux of the strain CEN.PK113-7D at 9 s spent in excess conditions over the course of cultivation.
- Figure S5: Growth, cell size and glycolytic flux of the strain Ethanol Red at 9 s spent in excess conditions over the course of cultivation.
- Figure S6: Growth, cell size and glycolytic flux of the strain Ethanol Red at 9 s spent in excess conditions over the course of cultivation.
- Figure S7: Growth, cell size and ATP level of the strain PE2 at 9 s spent in excess conditions over the course of cultivation.
- Figure S8: Growth, cell size and glycolytic flux of the strain PE2 at 9 s spent in excess conditions over the course of cultivation.
- Fig. S9 Adaption of growth and cell size of CEN.PK113-7D and PE2.
- Figure S10: Attempted Monod-kinetic fit to illustrate the growth curve behavior for all three yeast strains.
- Fig. S11: Heterogeneity of ATP levels within Ethanol Red.
- Fig. S12: Heatmaps of the distribution of ATP levels (QUEEN-2m ratio of uvGFP/GFP) over time for each tested condition and strain.
- Fig. S13: Heatmaps of the distribution of the GlyRNA sensor (ratio of CFP/RFP) as a proxy for glycolytic flux over time for each tested condition and strain.
- Fig. S14: Heatmaps of the distribution of cell size over time for each tested condition and strain.
- Figure S15: Population heterogeneity of QUEEN-2m (ATP levels), GlyRNA (FBP levels, glycolytic flux) and cell area in CEN.PK113-7D grown in dynamic glucose environments (23 s / 7s oscillation, top) and constant glucose environments (excess control, bottom).
- Figure S16: Population heterogeneity of QUEEN-2m (ATP levels), GlyRNA (FBP levels, glycolytic flux) and cell area in Ethanol Red grown in dynamic glucose environments (23 s / 7s oscillation, top) and constant glucose environments (excess control, bottom).
- Figure S17: Population heterogeneity of QUEEN-2m (ATP levels), GlyRNA (FBP levels, glycolytic flux) and cell area in PE2 grown in dynamic glucose environments (23 s / 7s oscillation, top) and constant glucose environments (excess control, bottom).

## Table S1

Tab. S1: Reference table for timescales in microbiology and bioprocessing.

| Duration / rate | | Process | Reference |
| --- | --- | --- | --- |
| From | To |  |  |
| 10 nt/s | 100 nt/s | Transcription | [1] |
| 10 aa/ s (~1 min/protein) |  | Translation | [1] |
| 1 ms | 1 min | Protein folding | [1] |
| 0.7 s (Fumarate in *EC*) | 3268 s (Ala in *SC*) | Metabolic turnover time | [2] |
| 1 s | 1 min | Metabolite pool half-life | [1] |
| 10 min | 10 hr | mRNA pool half-life | [1] |
| 1 h | 1 day | Protein pool half-life | [1] |
| 10 min (*VN*) | Days to years | Doubling time / µ | [3,4] |
| 200 generations (~1.4 days for *VN*) |  | Mutation accumulation / evolution | [5] |
| Few min | 2 – 10 h | Fluorophore maturation | [6] |
| 2-7 s | 5 - 15 s | Mixing time lab scale fermenter | [7,8] |
| 0.7 s | 40 s | Min/Max Regime residence time | [9] |
| 3.65 s | 9.37 s | Mean Regime residence time | [9] |
| 2.5* - 20 s | 77 s | Circulation time large scale fermenter (= mixing time / 4) | [9]  *Calculation from [8] |
| 10 s | 175 s | Mixing time 95 % homogeneity large scale | [8] |
|  | 100 generations | Fermentation time fed batch | [10] |
| 45-60 generations  (*VN* 7.5 h) |  | seed train to large scale (200 m^3^) | [10] |

Abbreviations: *Escherichia coli* (EC), *Saccharomyces cerevisiae* (SC), *Vibrio natriegens* (VN), nucleotides (nt), amino acids (aa)

References:

1. Shamir M, Bar-On Y, Phillips R, Milo R. SnapShot: Timescales in cell biology. Cell. 2016;164:1302-1302.e1. https://doi.org/10.1016/j.cell.2016.02.058

2. Wang G, Tang W, Xia J, Chu J, Noorman H, van Gulik WM. Integration of microbial kinetics and fluid dynamics toward model-driven scale-up of industrial bioprocesses. Eng Life Sci. 2015;15:20–9. https://doi.org/10.1002/elsc.201400172

3. Hoff J, Daniel B, Stukenberg D, Thuronyi BW, Waldminghaus T, Fritz G. Vibrio natriegens: an ultrafast-growing marine bacterium as emerging synthetic biology chassis. Environ Microbiol. 2020;22:4394–408. https://doi.org/10.1111/1462-2920.15128

4. Weissman JL, Hou S, Fuhrman JA. Estimating maximal microbial growth rates from cultures, metagenomes, and single cells via codon usage patterns. Proc Natl Acad Sci. Proceedings of the National Academy of Sciences; 2021;118:e2016810118. https://doi.org/10.1073/pnas.2016810118

5. Nguyen J, Lara-Gutiérrez J, Stocker R. Environmental fluctuations and their effects on microbial communities, populations and individuals. FEMS Microbiol Rev. 2021;45. https://doi.org/10.1093/femsre/fuaa068

6. Balleza E, Kim JM, Cluzel P. Systematic characterization of maturation time of fluorescent proteins in living cells. Nat Methods. Nature Publishing Group; 2018;15:47–51. https://doi.org/10.1038/nmeth.4509

7. Gaugler L, Hofmann S, Schlüter M, Takors R. Mimicking CHO large-scale effects in the single multicompartment bioreactor: A new approach to access scale-up behavior. Biotechnol Bioeng. 2024;121:1243–55. https://doi.org/10.1002/bit.28647

8. Lara AR, Galindo E, Ramírez OT, Palomares LA. Living with heterogeneities in bioreactors: Understanding the effects of environmental gradients on cells. Mol Biotechnol. 2006;34:355–82. https://doi.org/10.1385/MB:34:3:355

9. Haringa C, Tang W, Deshmukh AT, Xia J, Reuss M, Heijnen JJ, et al. Euler-Lagrange computational fluid dynamics for (bio)reactor scale down: An analysis of organism lifelines. Eng Life Sci. 2016;16:652–63. https://doi.org/10.1002/elsc.201600061

10. Rugbjerg P, Myling-Petersen N, Porse A, Sarup-Lytzen K, Sommer MOA. Diverse genetic error modes constrain large-scale bio-based production. Nat Commun. Nature Publishing Group; 2018;9:787. https://doi.org/10.1038/s41467-018-03232-w

## Figure S1


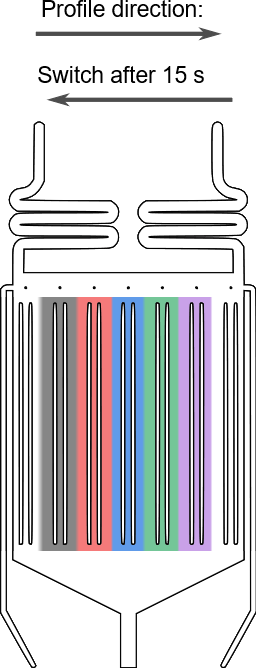

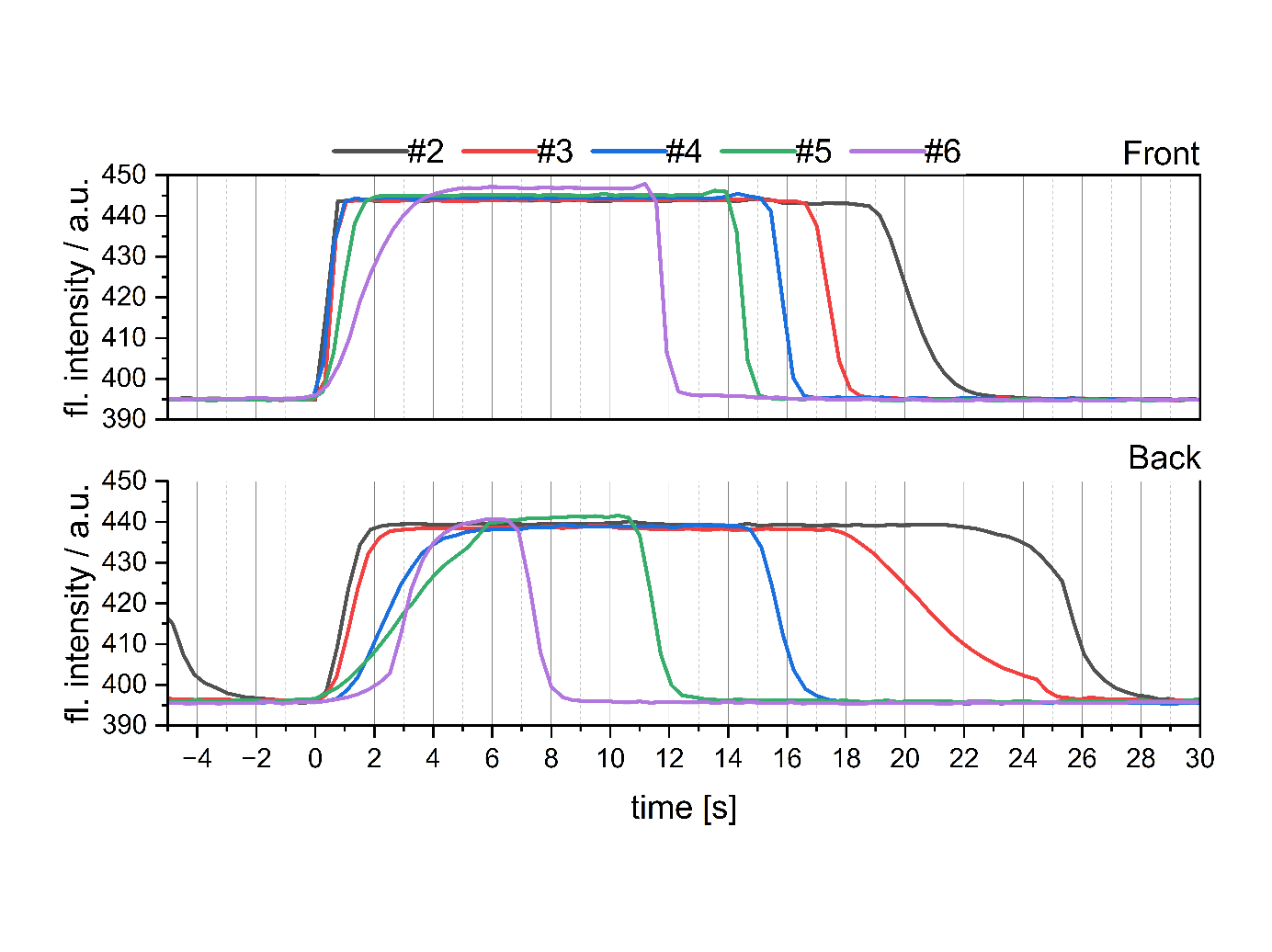


Fig. S1: Characterization of the applied microfluidic chip concerning flow profile definition.

Fluorescent dye exchange in 80 × 90 × 4 µm MGCs was analyzed as a function of chamber position (see Materials and Methods in main paper). For each array pair (#2–#6) within the switching zone, chambers located at both the front and the rear of the array were selected. Pressure conditions were alternated every 15 s, with one representative switching cycle shown. This induced a lateral displacement of the laminar interface from left to right, followed by a return after 15 s. To account for switching-induced delays, time was normalized to the onset of the first detectable signal change within each chamber, thereby excluding the profile switching time from the analysis.

Upon pressure switching, the laminar interface requires 2.7 s to traverse across the whole switching zone, resulting in sequential exposure of array pairs and deviations from the intended 15 s per condition, with only the central array pair #4 experiencing the programmed profile. Exposure times vary substantially across the device, increasing toward the outer arrays and reaching up to 22.5 s deviation in array pair #2. Medium exchange within individual MGCs is diffusion-driven and position-dependent, with slower exchange at the rear of arrays due to gradually developing concentration gradients caused by downstream convection.

## Figure S2


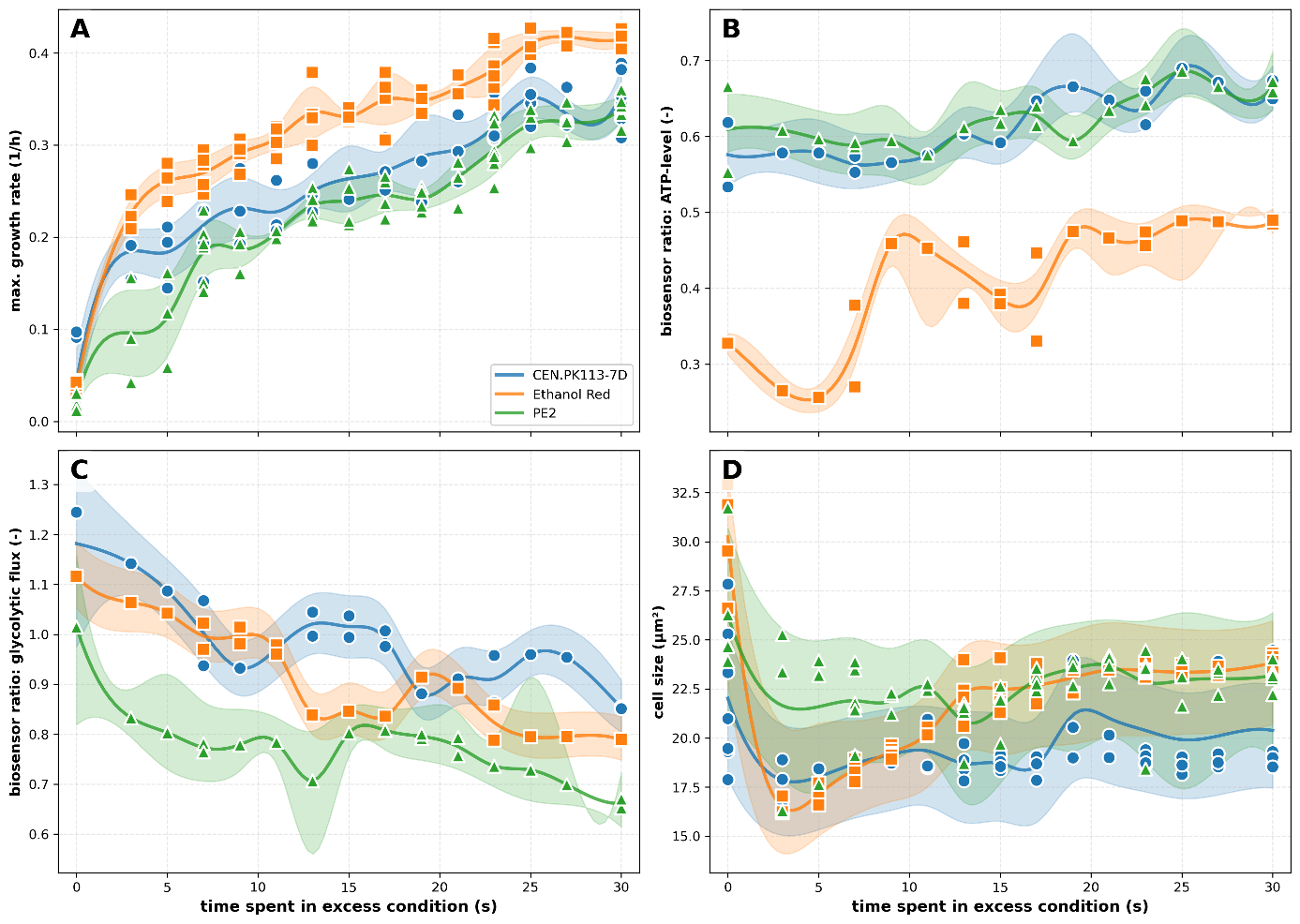


Fig. S2: Cellular performance of the three examined yeast strains for different times spent in excess condition.

The figure shows different key performance indicators (KPIs) of the three yeast strains investigated. The maximum growth rate (µ_max_) was determined at the population level (A). Cellular ATP levels and glycolytic flux were assessed using the QUEEN-2m sensor (B) and GlyRNA sensor (C), while cell size was monitored in parallel (D). For panels B–D, data are presented as the median of all single-cell measurements ± interquartile range (25–75%) collected over 10–18 hours of cultivation. When multiple experiments were performed under the same dynamic condition, spline curves were generated based on the means of the medians.

## Figure S3


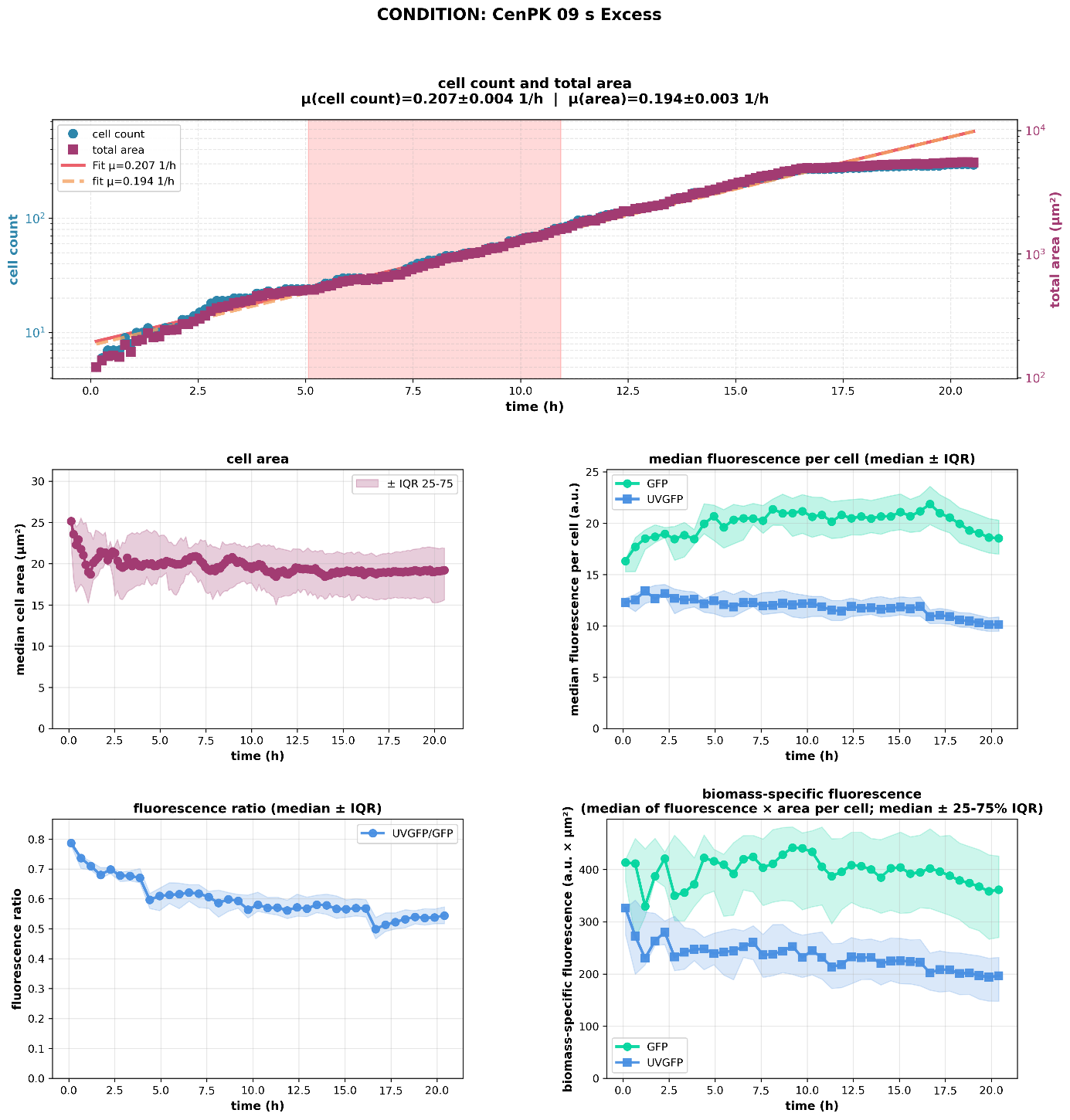


Fig. S3: Growth, cell size and ATP level of the strain CEN.PK113-7D at 9 s spent in excess conditions over the course of cultivation.

The panel illustrates the temporal development of KPIs throughout the cultivation. Cell count and total colony area were used as proxies for cellular growth, with linear fits applied to both parameters to estimate growth rates. Single-cell area served as an indicator of cell size and morphological changes. Biosensor dynamics were assessed based on the fluorescence intensities of two distinct fluorescence channels, representing different conformational states of the QUEEN-2m sensor. The ratio of these fluorescence signals was used to track changes in intracellular ATP levels over the course of cultivation, while biomass-specific fluorescence signals were derived by normalizing fluorescence intensities to the cell area. For all panels excluding the cell count/total area, data are presented as the median of all single-cell measurements ± interquartile range (25–75%) collected over the course of cultivation.

## Figure S4


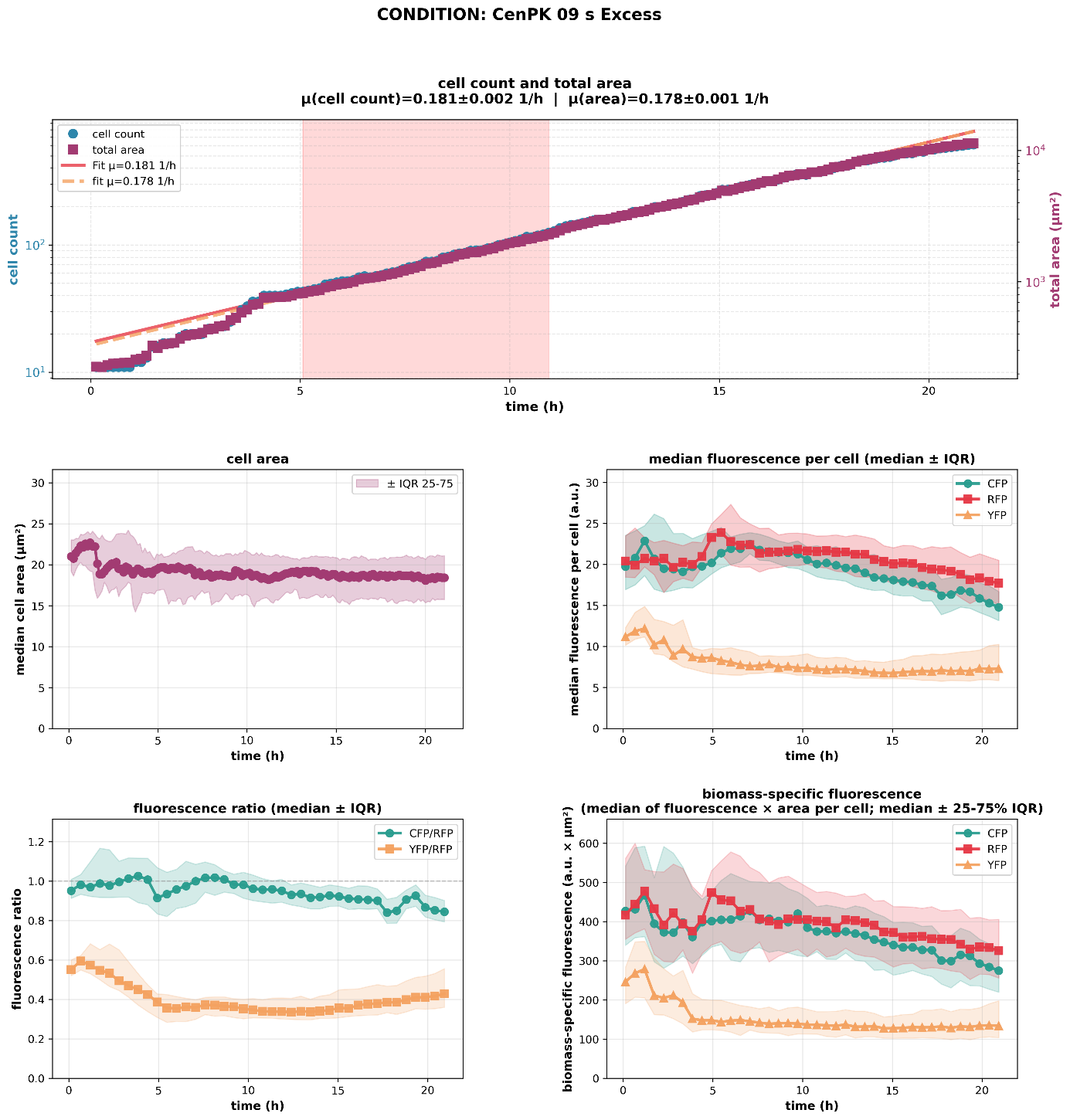


Fig. S4: Growth, cell size and glycolytic flux of the strain CEN.PK113-7D at 9 s spent in excess conditions over the course of cultivation.

The panel illustrates the temporal development of KPIs throughout the cultivation. Cell count and total colony area were used as proxies for cellular growth, with linear fits applied to both parameters to estimate growth rates. Single-cell area served as an indicator of cell size and morphological changes. Biosensor dynamics were assessed based on the ratio the fluorescence intensities of two distinct fluorophores. CFP levels correlate reversely with fructose-1,6-bisphosphate levels and the signal are normalised to constitutively expressed RFP. The ratio of these fluorescence signals was used to track changes in the glycolytic flux over the course of cultivation, while biomass-specific fluorescence signals were derived by normalizing fluorescence intensities to the cell area. The displayed YFP signal and resulting YFP/RFP ratio indicate the level of oxidative stress but was not investigated in the presented study. For all panels excluding the cell count/total area, data are presented as the median of all single-cell measurements ± interquartile range (25–75%) collected over the course of cultivation.

## Figure S5


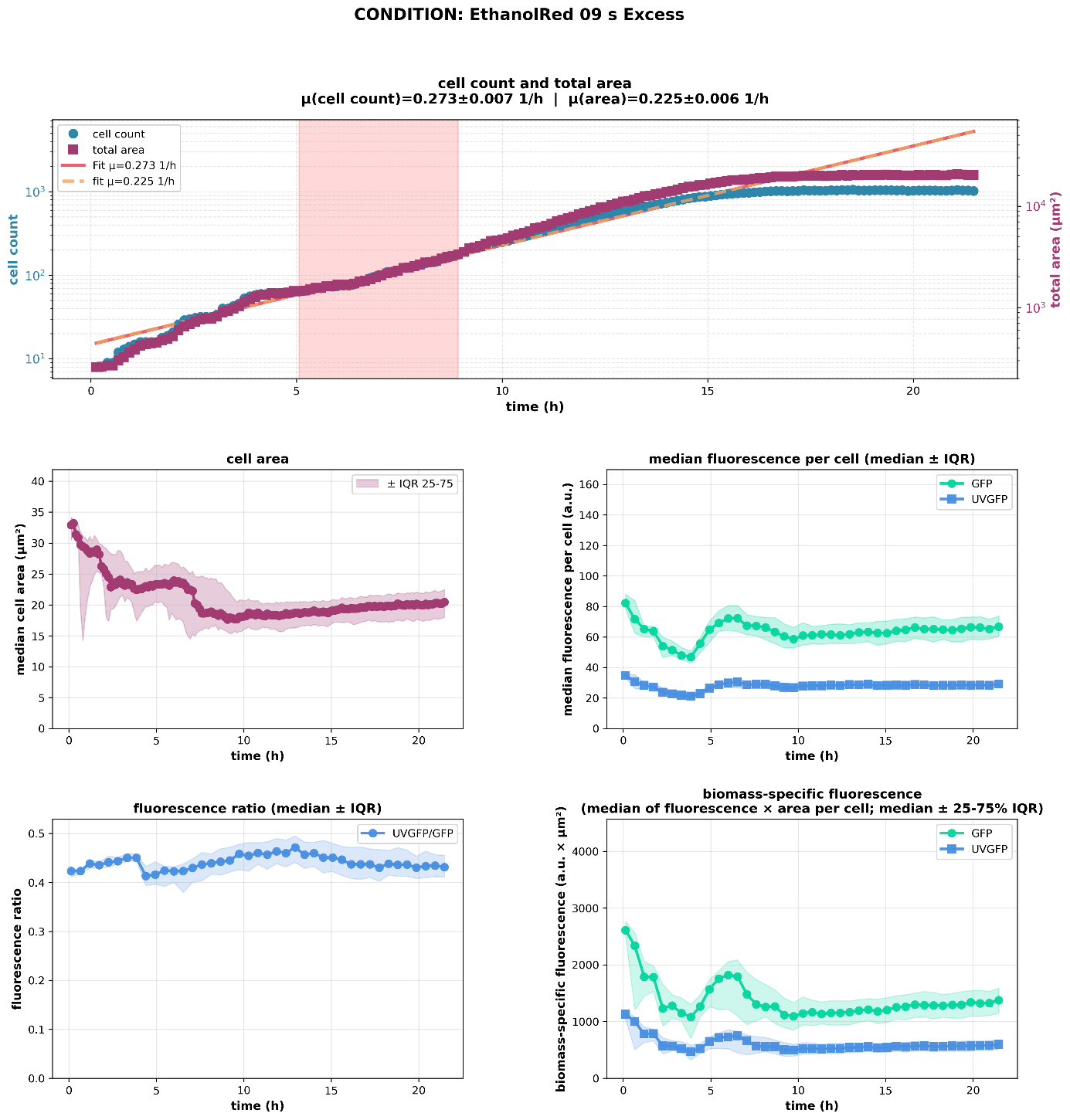


Fig. S5: Growth, cell size and ATP level of the strain Ethanol Red at 9 s spent in excess conditions over the course of cultivation.

The panel illustrates the temporal development of KPIs throughout the cultivation. Cell count and total colony area were used as proxies for cellular growth, with linear fits applied to both parameters to estimate growth rates. Single-cell area served as an indicator of cell size and morphological changes. Biosensor dynamics were assessed based on the fluorescence intensities of two distinct fluorescence channels, representing different conformational states of the QUEEN-2m sensor. The ratio of these fluorescence signals was used to track changes in intracellular ATP levels over the course of cultivation, while biomass-specific fluorescence signals were derived by normalizing fluorescence intensities to the cell area. For all panels excluding the cell count/total area, data are presented as the median of all single-cell measurements ± interquartile range (25–75%) collected over the course of cultivation.

## Figure S6


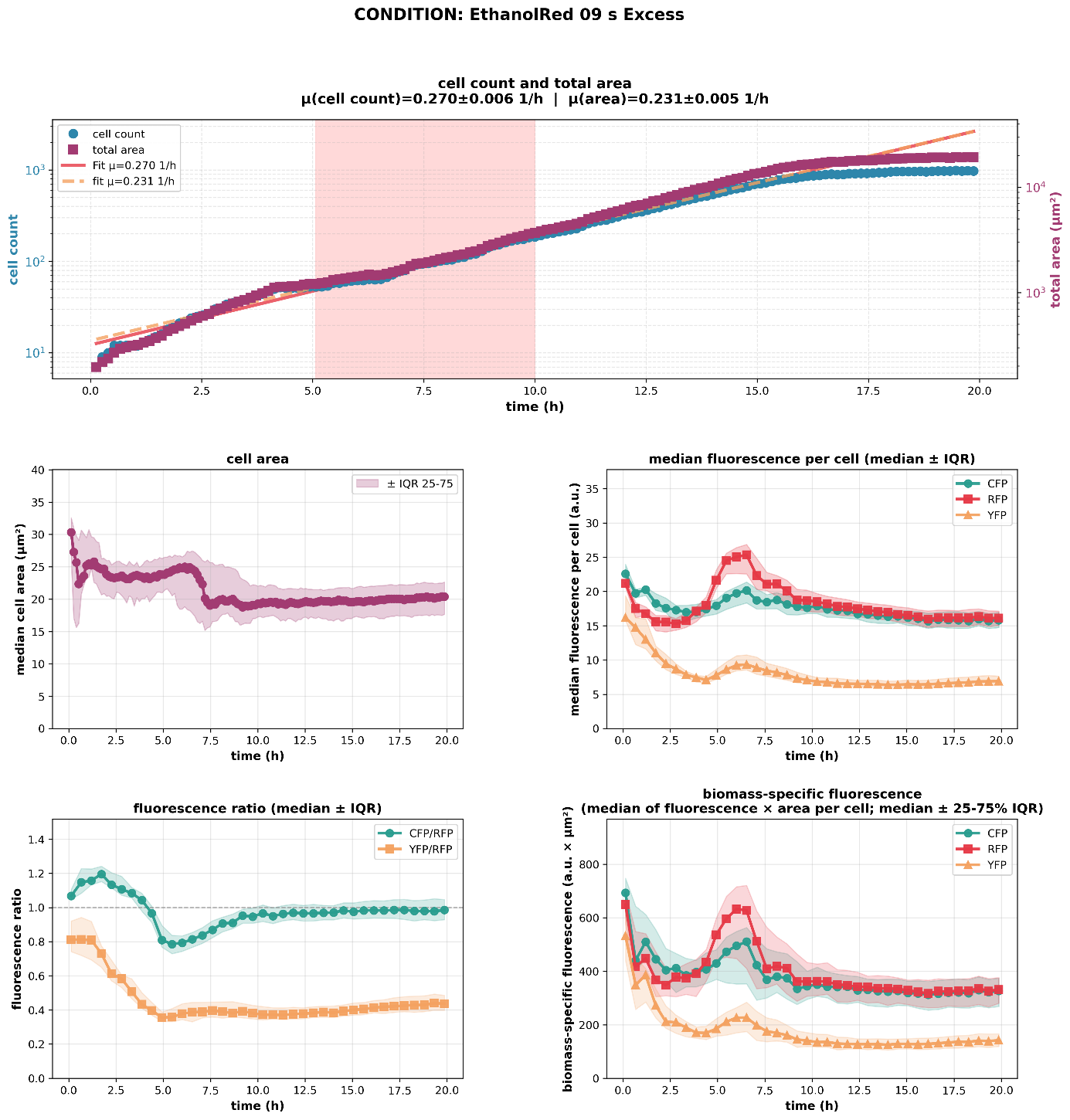


Fig. S6: Growth, cell size and glycolytic flux of the strain Ethanol Red at 9 s spent in excess conditions over the course of cultivation.

The panel illustrates the temporal development of KPIs throughout the cultivation. Cell count and total colony area were used as proxies for cellular growth, with linear fits applied to both parameters to estimate growth rates. Single-cell area served as an indicator of cell size and morphological changes. Biosensor dynamics were assessed based on the ratio the fluorescence intensities of two distinct fluorophores. CFP levels correlate reversely with fructose-1,6-bisphosphate levels and the signal are normalised to constitutively expressed RFP. The ratio of these fluorescence signals was used to track changes in the glycolytic flux over the course of cultivation, while biomass-specific fluorescence signals were derived by normalizing fluorescence intensities to the cell area. The displayed YFP signal and resulting YFP/RFP ratio indicate the level of oxidative stress but was not investigated in the presented study. For all panels excluding the cell count/total area, data are presented as the median of all single-cell measurements ± interquartile range (25–75%) collected over the course of cultivation.

## Figure S7


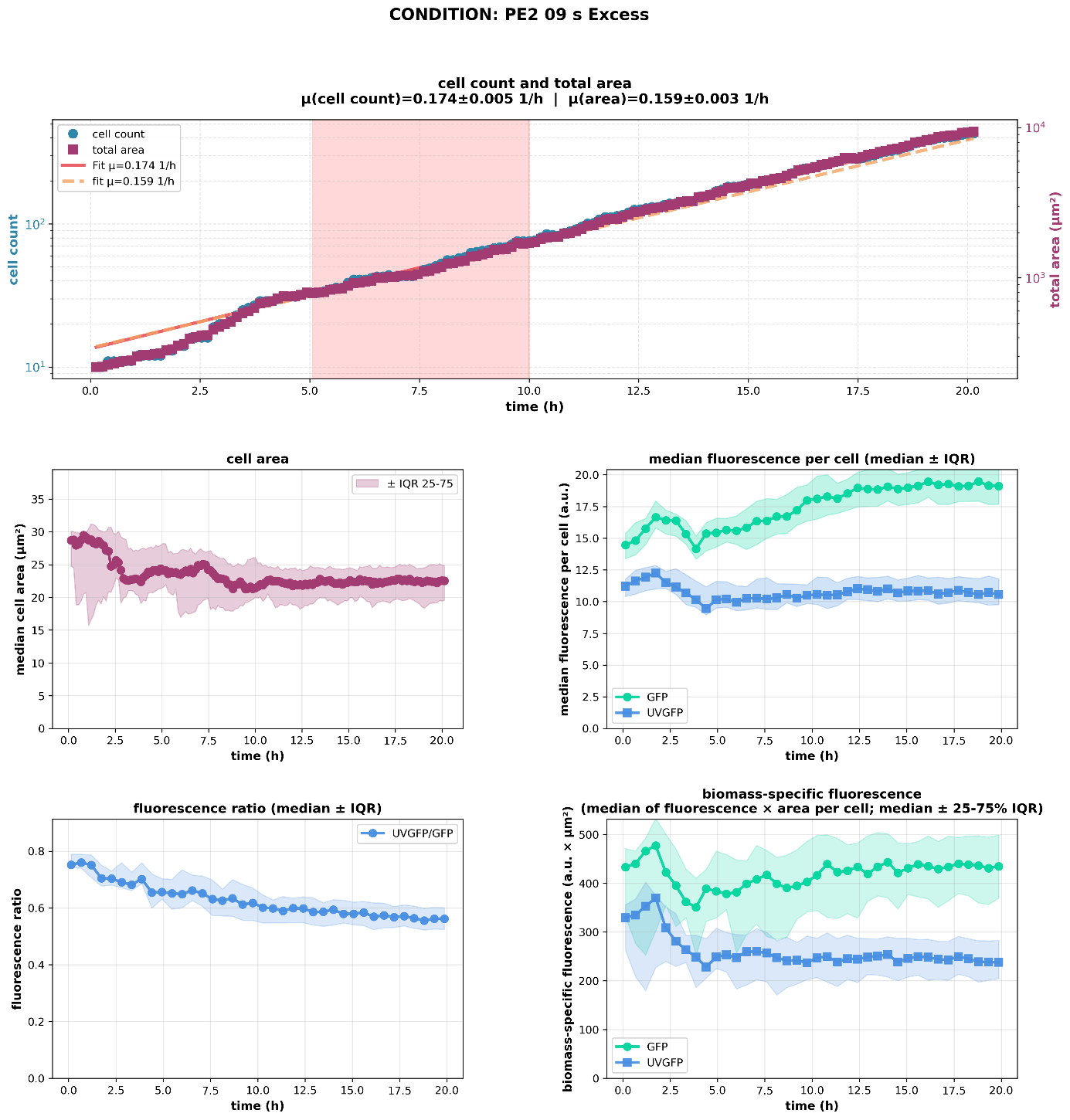


Fig. S7: Growth, cell size and ATP level of the strain PE2 at 9 s spent in excess conditions over the course of cultivation.

The panel illustrates the temporal development of KPIs throughout the cultivation. Cell count and total colony area were used as proxies for cellular growth, with linear fits applied to both parameters to estimate growth rates. Single-cell area served as an indicator of cell size and morphological changes. Biosensor dynamics were assessed based on the fluorescence intensities of two distinct fluorescence channels, representing different conformational states of the QUEEN-2m sensor. The ratio of these fluorescence signals was used to track changes in intracellular ATP levels over the course of cultivation, while biomass-specific fluorescence signals were derived by normalizing fluorescence intensities to the cell area. For all panels excluding the cell count/total area, data are presented as the median of all single-cell measurements ± interquartile range (25–75%) collected over the course of cultivation.

## Figure S8


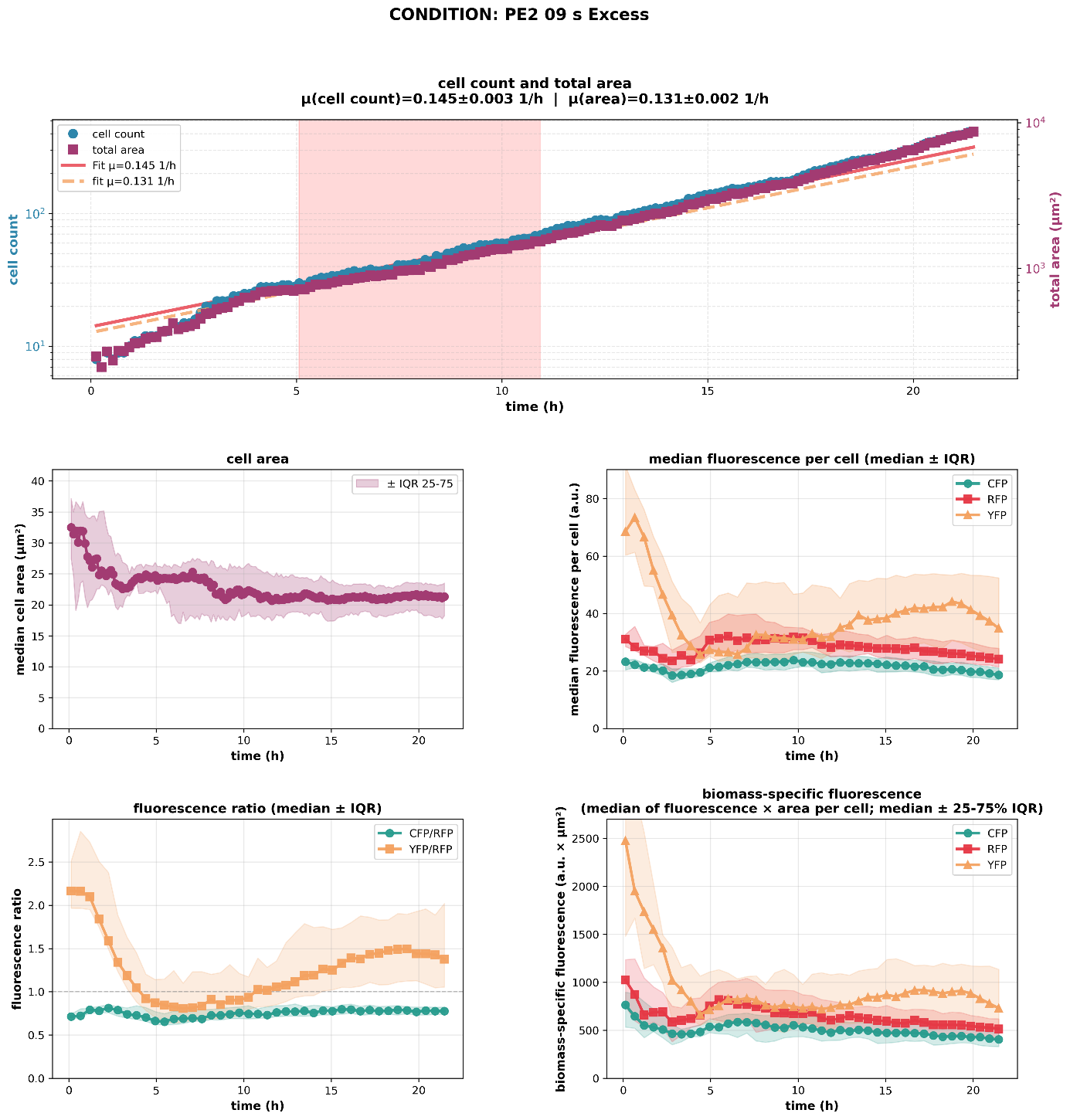


Fig. S8: Growth, cell size and glycolytic flux of the strain PE2 at 9 s spent in excess conditions over the course of cultivation.

The panel illustrates the temporal development of KPIs throughout the cultivation. Cell count and total colony area were used as proxies for cellular growth, with linear fits applied to both parameters to estimate growth rates. Single-cell area served as an indicator of cell size and morphological changes. Biosensor dynamics were assessed based on the ratio the fluorescence intensities of two distinct fluorophores. CFP levels correlate reversely with fructose-1,6-bisphosphate levels and the signal are normalised to constitutively expressed RFP. The ratio of these fluorescence signals was used to track changes in the glycolytic flux over the course of cultivation, while biomass-specific fluorescence signals were derived by normalizing fluorescence intensities to the cell area. The displayed YFP signal and resulting YFP/RFP ratio indicate the level of oxidative stress but was not investigated in the presented study. For all panels excluding the cell count/total area, data are presented as the median of all single-cell measurements ± interquartile range (25–75%) collected over the course of cultivation.

## Figure S9

| **CEN.PK113-7D** | **PE2** |
| --- | --- |
| 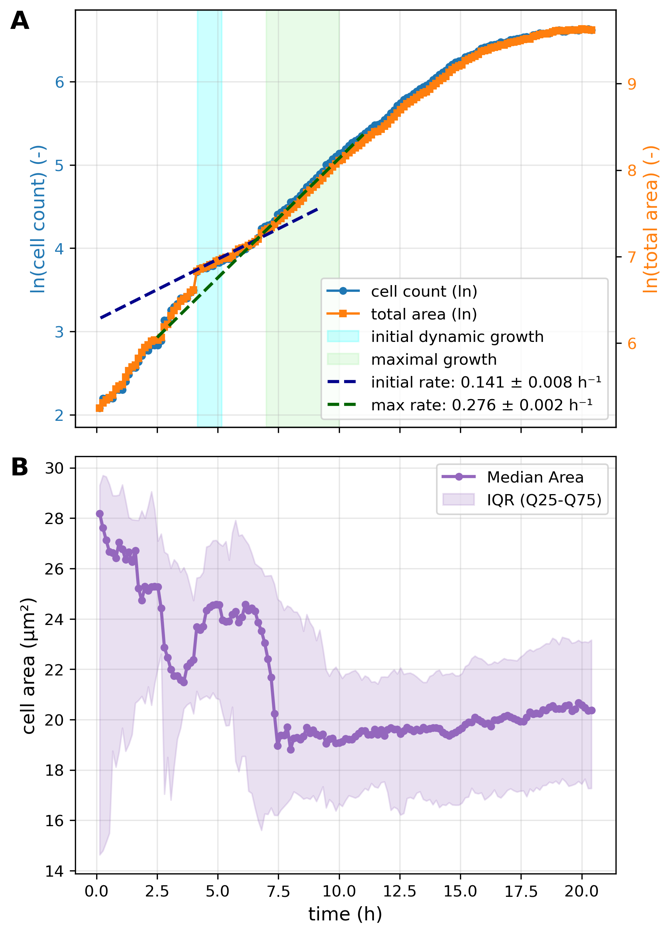 | 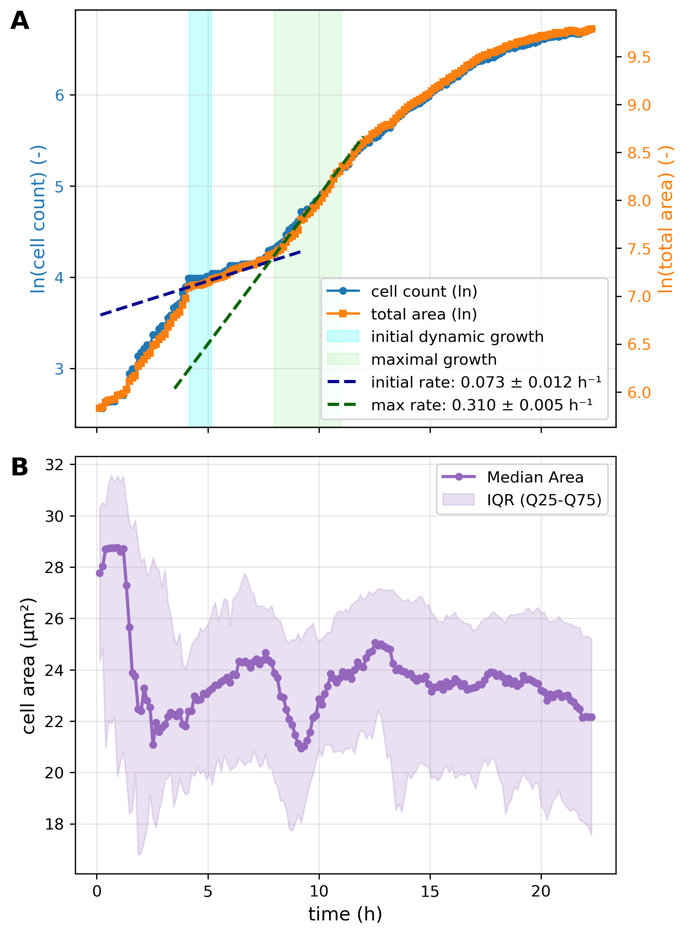 |

Fig. S_X8 Adaption of growth and cell size of CEN.PK113-7D and PE2.

(A) Growth curve of respective strain with 9 s spent in excess (9 s/21 s oscillation) based on cell count and total colony area. The initial growth rate (µ_ini_) was calculated over the first hour after the onset of the dynamics at 4 h cultivation time (blue), while the timeframe for µ_max_ is marked in green. (B) Cell size development of cultivation in A showing the median cell area and the interquartile range. Data for Ethanol Red can be found in the main manuscript, Fig. 5.

## Figure S10


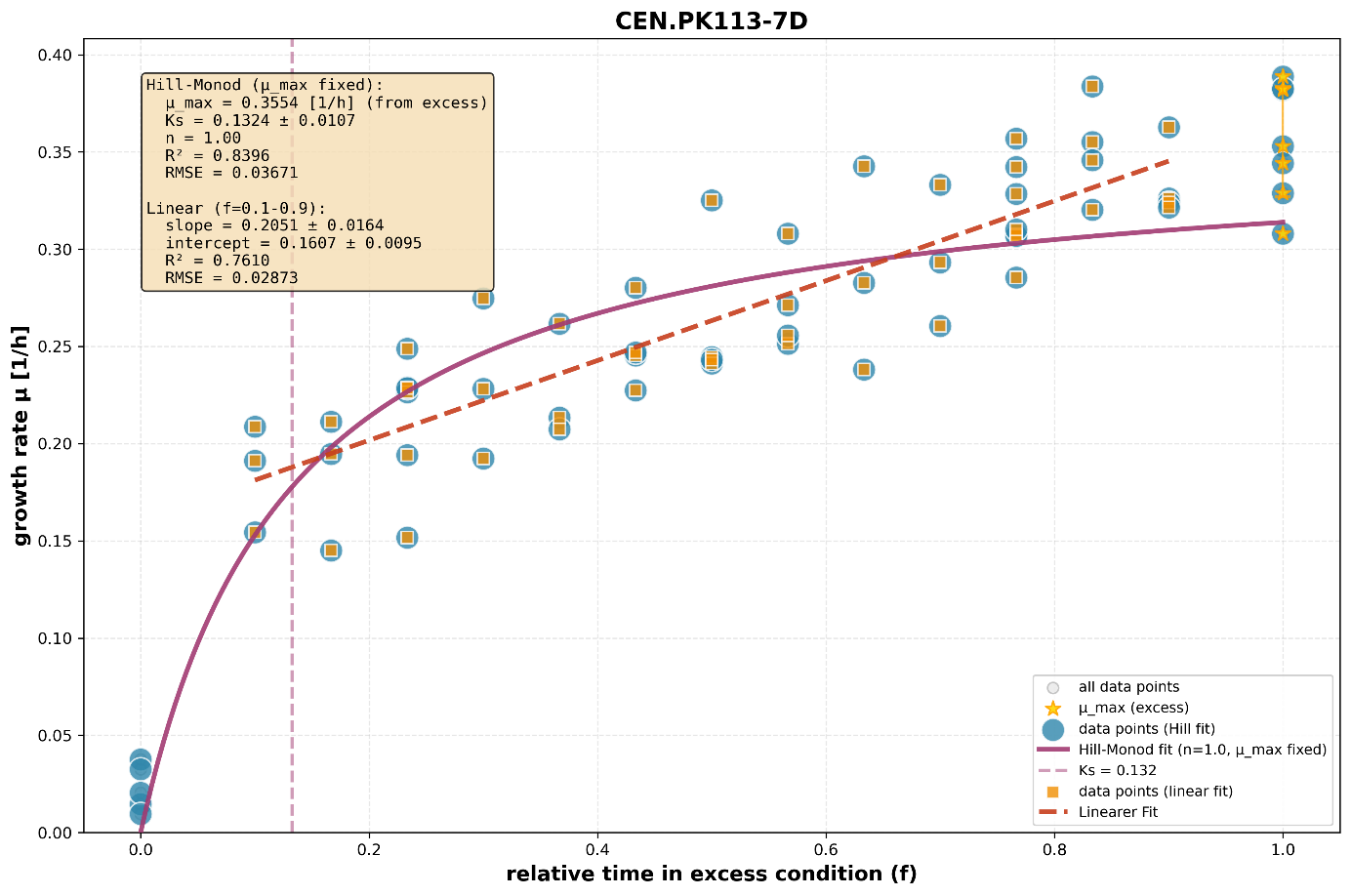

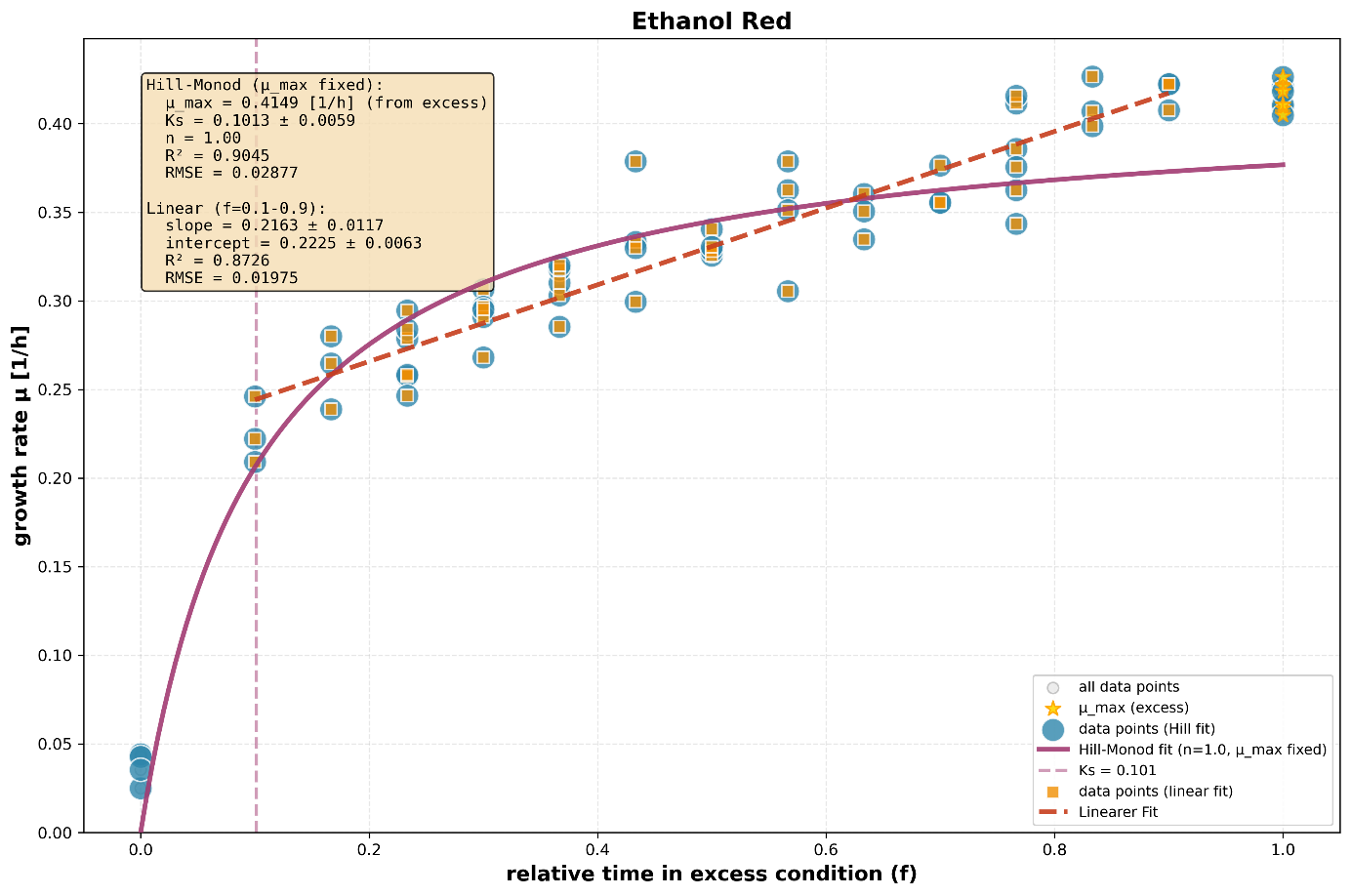


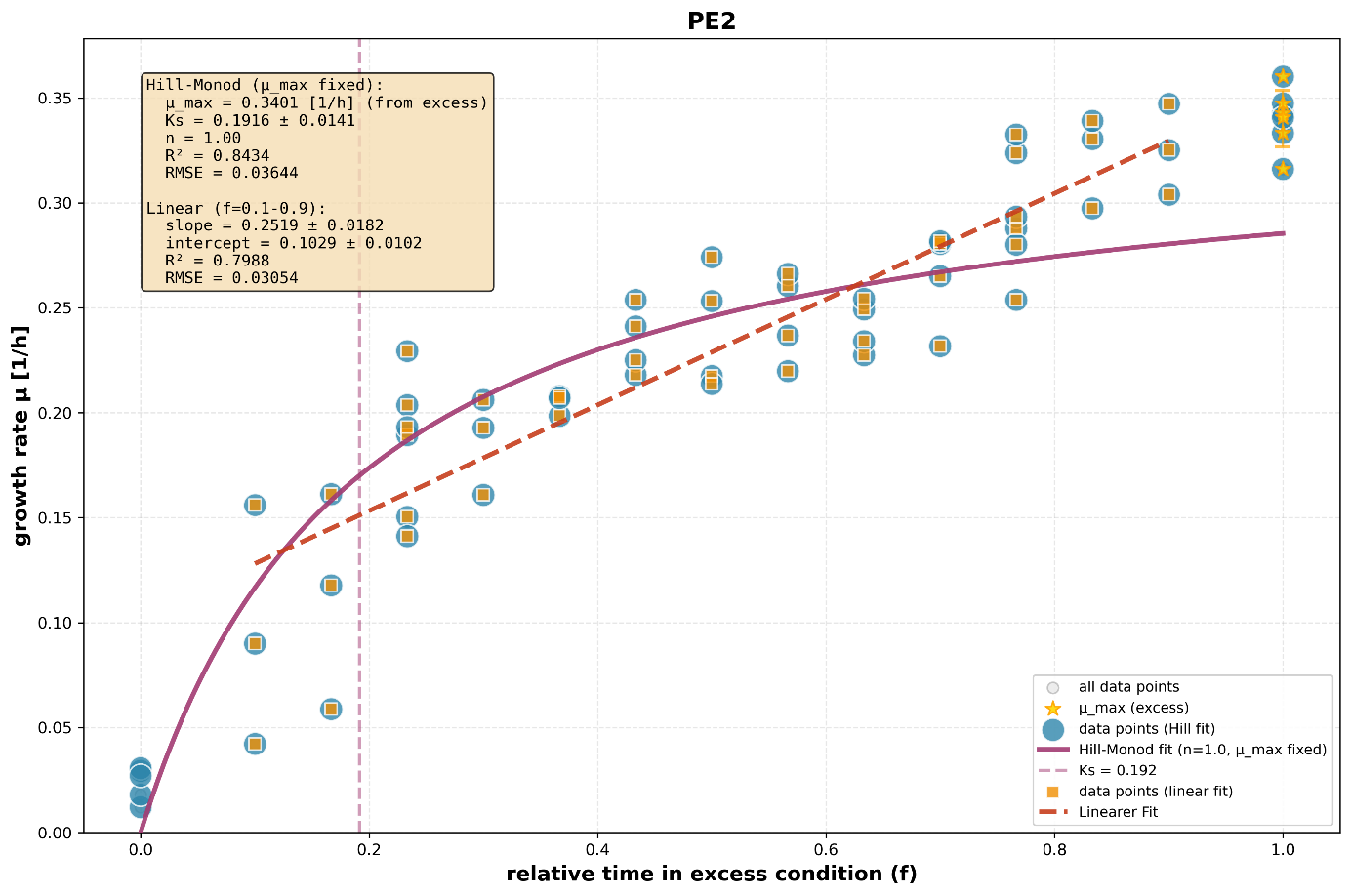


Fig. S9: Attempted Monod-kinetic fit and linear fit to illustrate the growth curve behavior for all three yeast strains.

## Figure S11

| **A** | 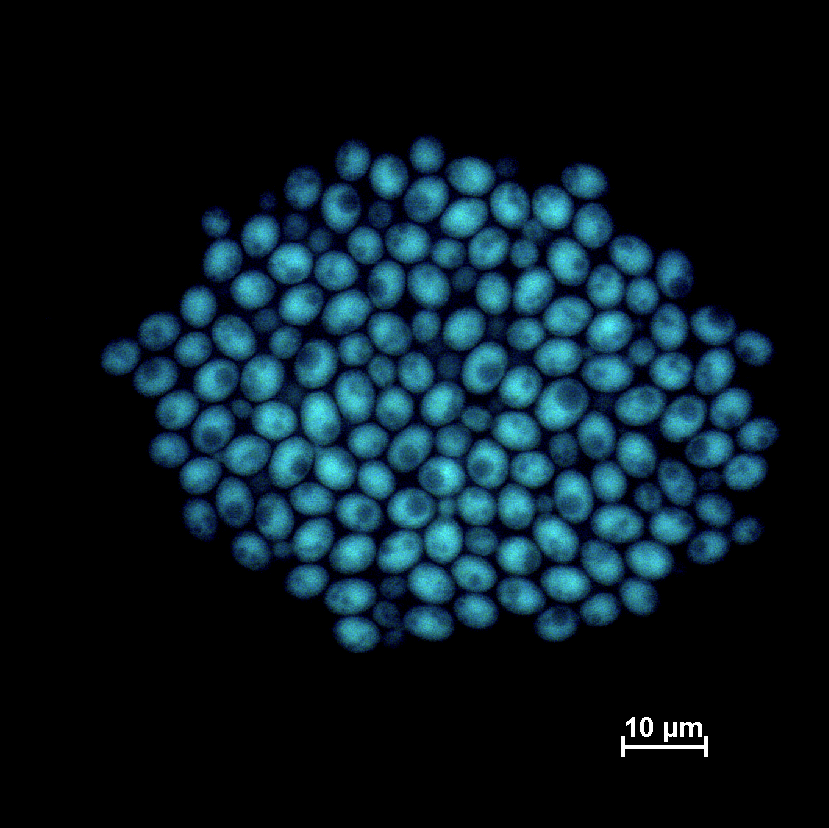  **FoV 1** | **B** | 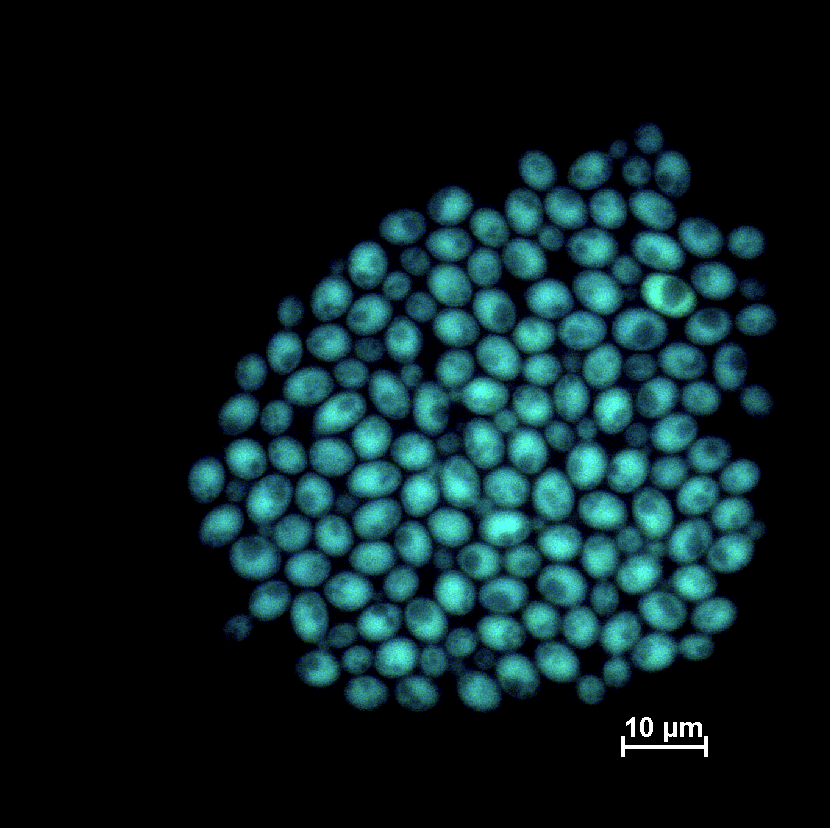  **FoV 3** |
| --- | --- | --- | --- |
| **C**  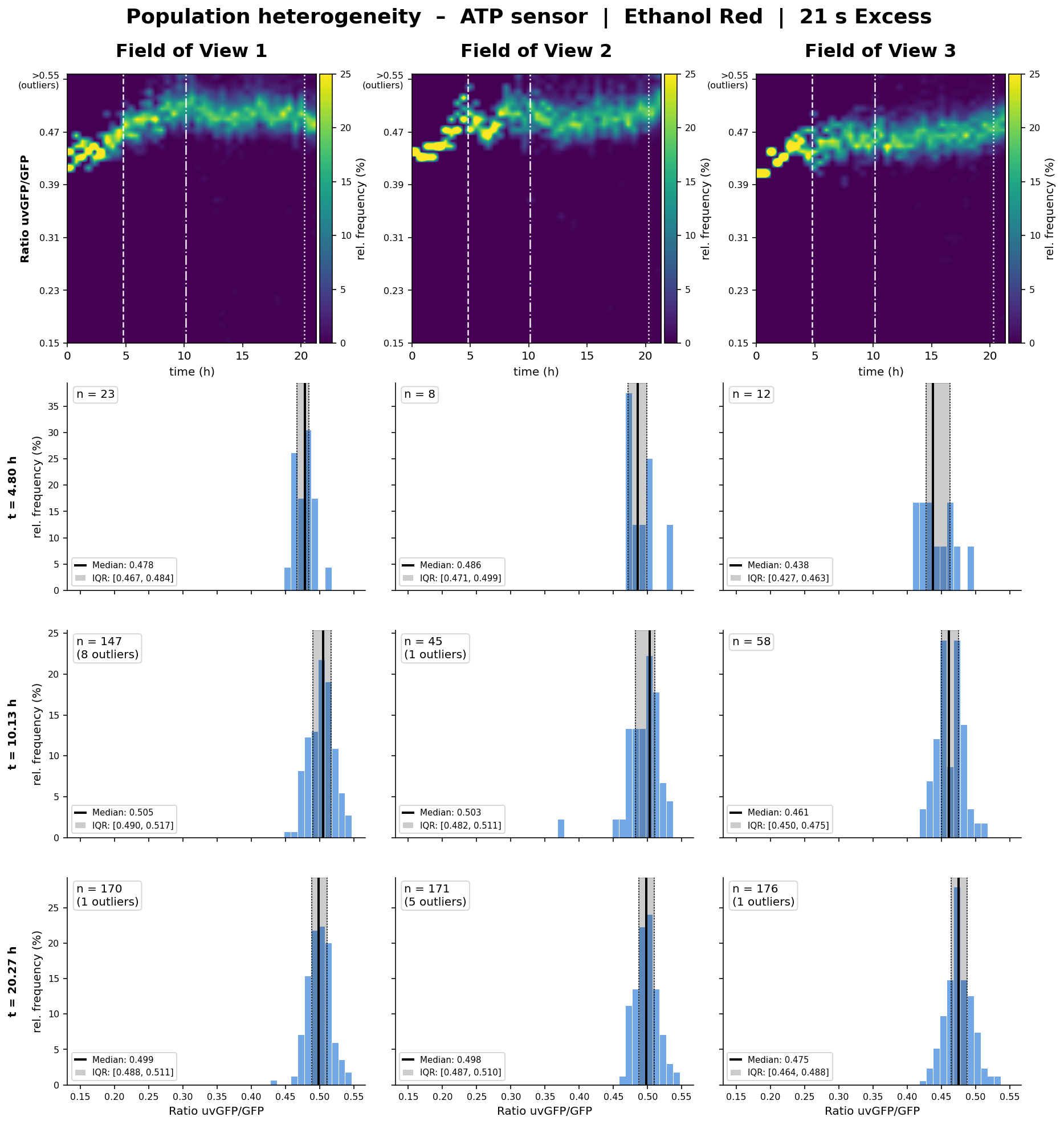 | | | |

Fig. S11: Heterogeneity of ATP levels within Ethanol Red.

Panel A and B show the ratiometric biosensor signal of QUEEN-2m for two fields of view (FoV) within the same experiment of Ethanol Red (21 s spent in glucose excess (21 s/9 s oscillation). The microscopic images are an overlay of the uvgfp and the gfp signal, each displayed with the same LUTs (uvgfp: 430 – 490, gfp: 475 – 591) to show the difference in “greenness” between cells.

Panel C shows the heatmap of QUEEN-2m ratio over time for three FoVs within the same experiment. Furthermore, the histograms of these FoVs at three different timepoints are shown and the median and IQR are calculated. Two-sided Mann-Whitney-U-test was calculated for each timepoint, to test for significant differences between the population in each FoV. FoV 3 is statistically significantly different to FoV 1 and 2 for each timepoint.

Mann-Whitney-U-Test:

t = 4.800 h

-------------------------------------------------------

FOV 1: n=23, Median=0.4781, IQR=[0.4668, 0.4844]

FOV 2: n=8, Median=0.4860, IQR=[0.4713, 0.4988]

FOV 3: n=12, Median=0.4378, IQR=[0.4273, 0.4628]

FOV1 vs FOV2: U=63.0, p=2.0261e-01 n.s.

FOV1 vs FOV3: U=237.0, p=6.1909e-04 ***

FOV2 vs FOV3: U=90.0, p=4.7630e-04 ***

t = 10.133 h

-------------------------------------------------------

FOV 1: n=147, Median=0.5051, IQR=[0.4897, 0.5170]

FOV 2: n=45, Median=0.5027, IQR=[0.4821, 0.5110]

FOV 3: n=58, Median=0.4608, IQR=[0.4504, 0.4755]

FOV1 vs FOV2: U=3918.0, p=6.1463e-02 n.s.

FOV1 vs FOV3: U=7987.0, p=2.1860e-22 ***

FOV2 vs FOV3: U=2298.0, p=4.1368e-11 ***

t = 20.267 h

-------------------------------------------------------

FOV 1: n=170, Median=0.4988, IQR=[0.4884, 0.5107]

FOV 2: n=171, Median=0.4983, IQR=[0.4874, 0.5097]

FOV 3: n=176, Median=0.4748, IQR=[0.4641, 0.4881]

FOV1 vs FOV2: U=14946.0, p=6.5200e-01 n.s.

FOV1 vs FOV3: U=24725.0, p=8.8489e-26 ***

FOV2 vs FOV3: U=24428.0, p=1.0185e-23 ***

## Figure S12

##
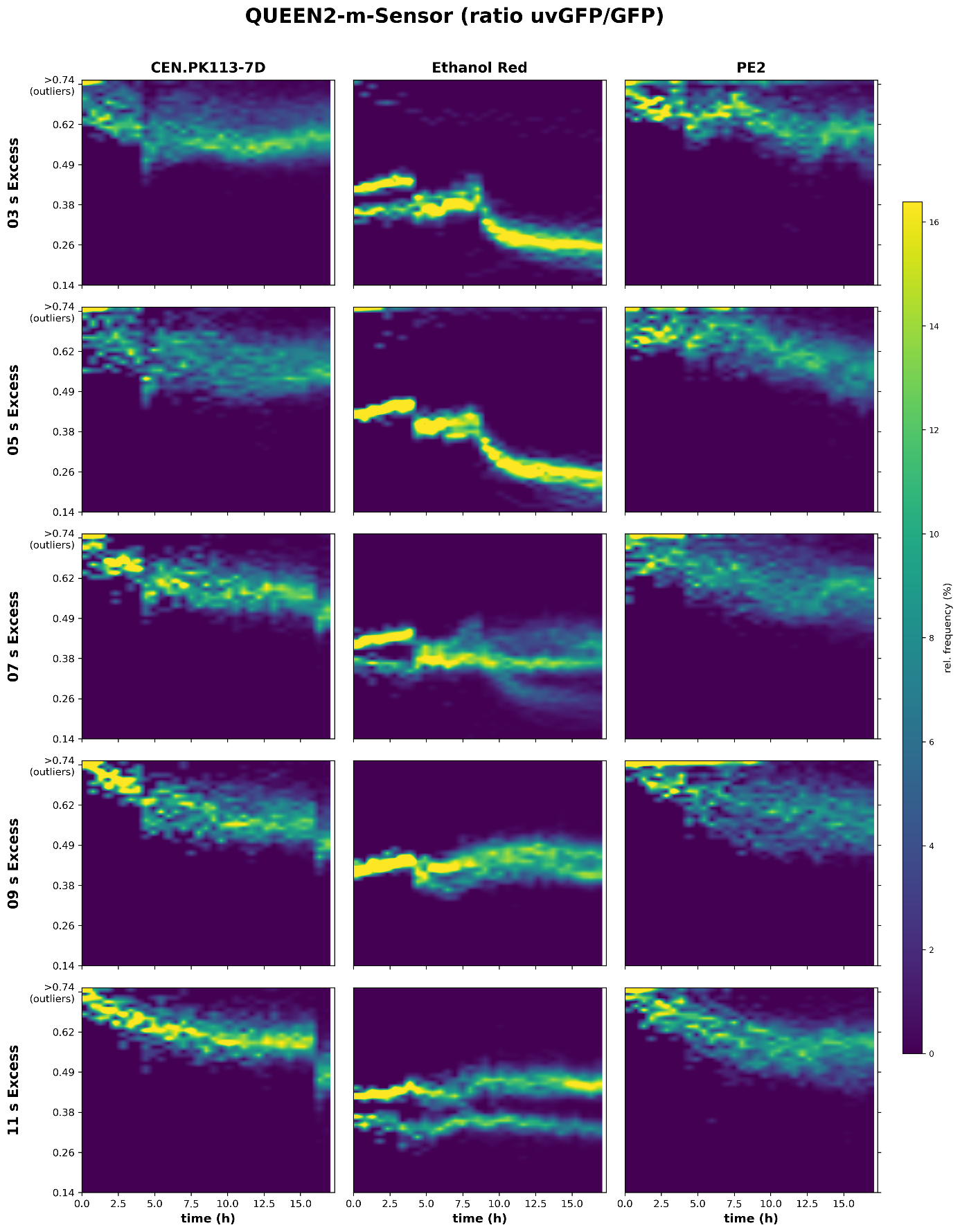


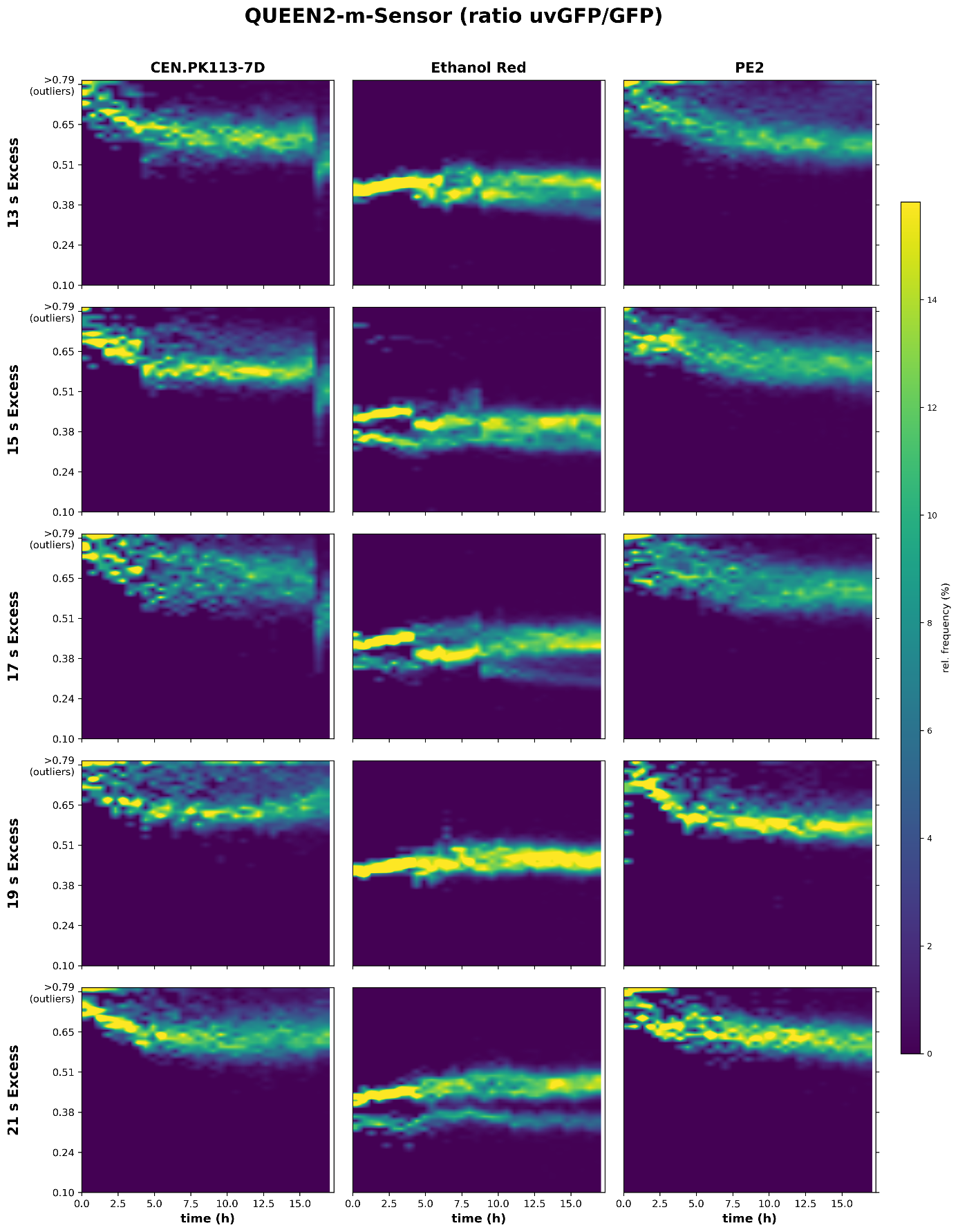


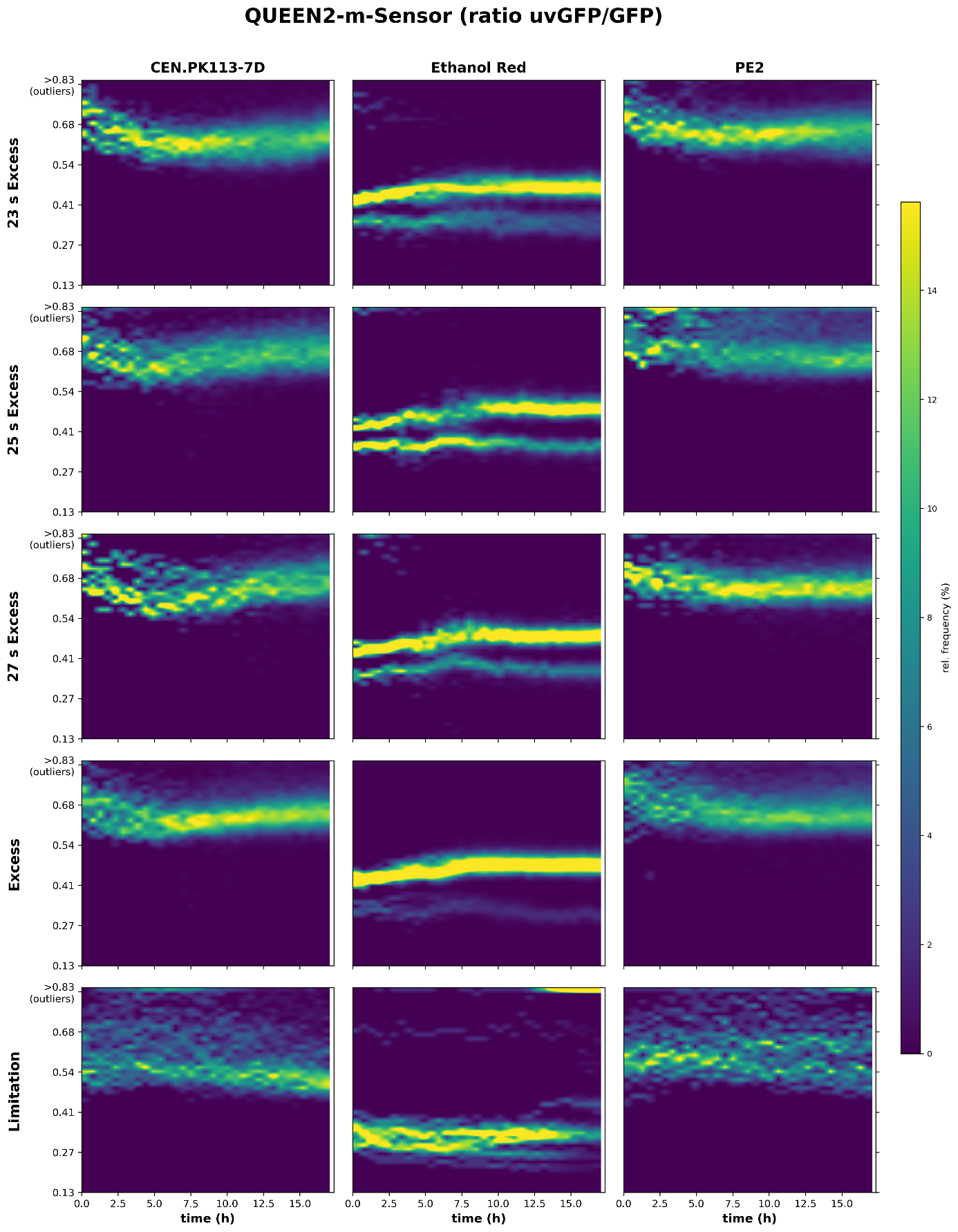


Fig. S12: Heatmaps of the distribution of ATP levels (QUEEN-2m ratio of uvGFP/GFP) over time for each tested condition and strain.

One heatmap includes the single-cell data of all recorded FoVs for that strain and condition. The cultivation duration was cropped to the one of the shortest experiment.

## Figure S13


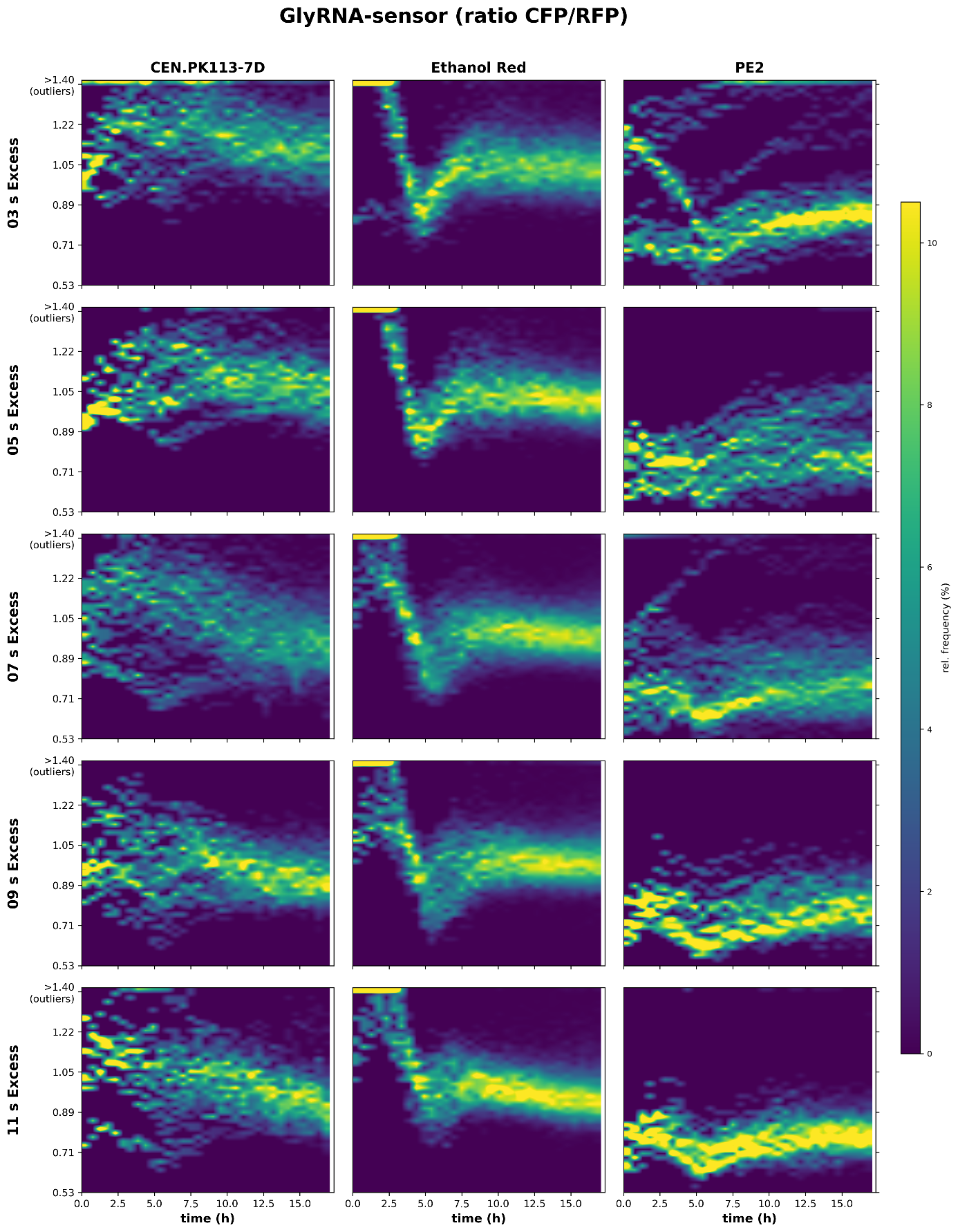


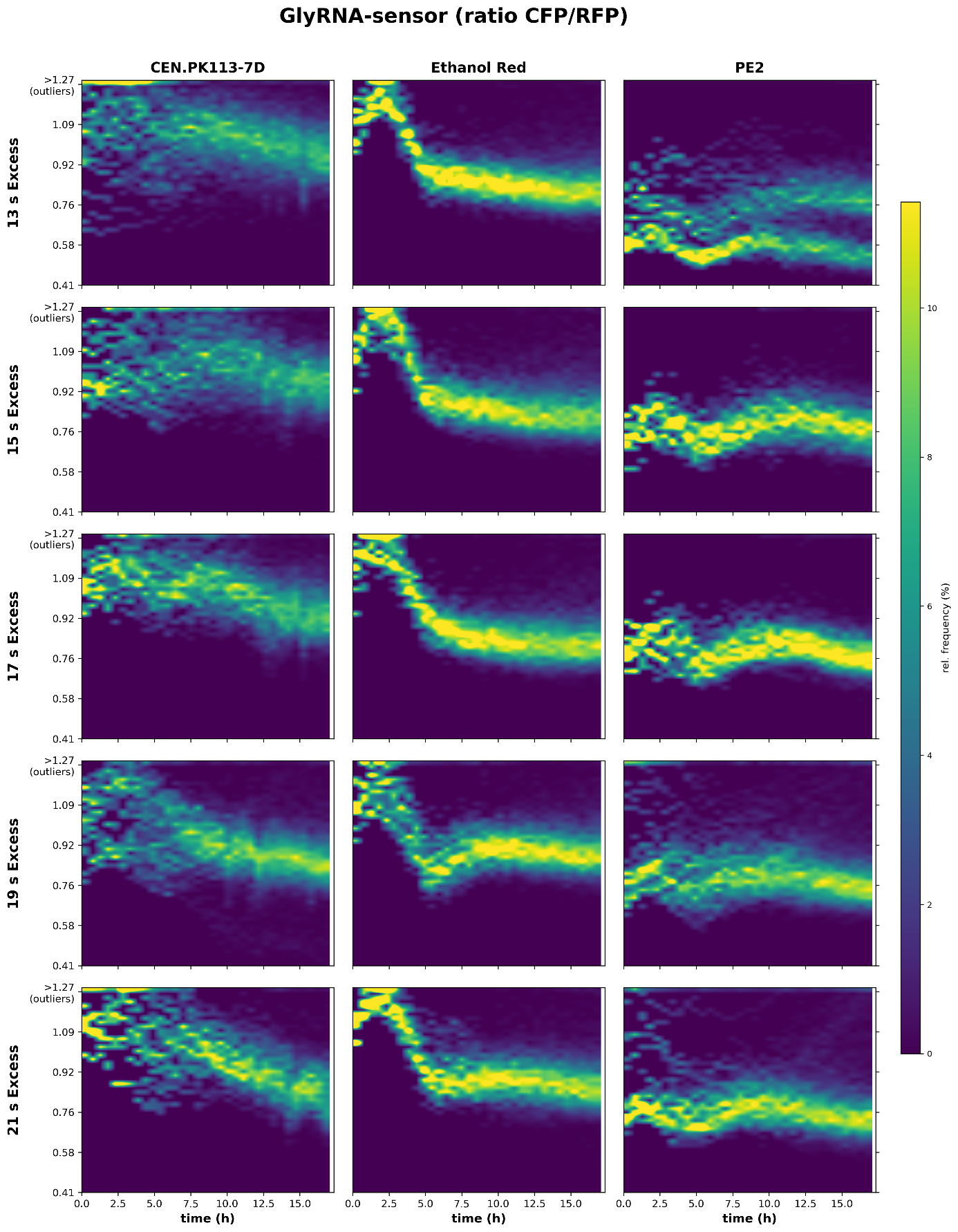


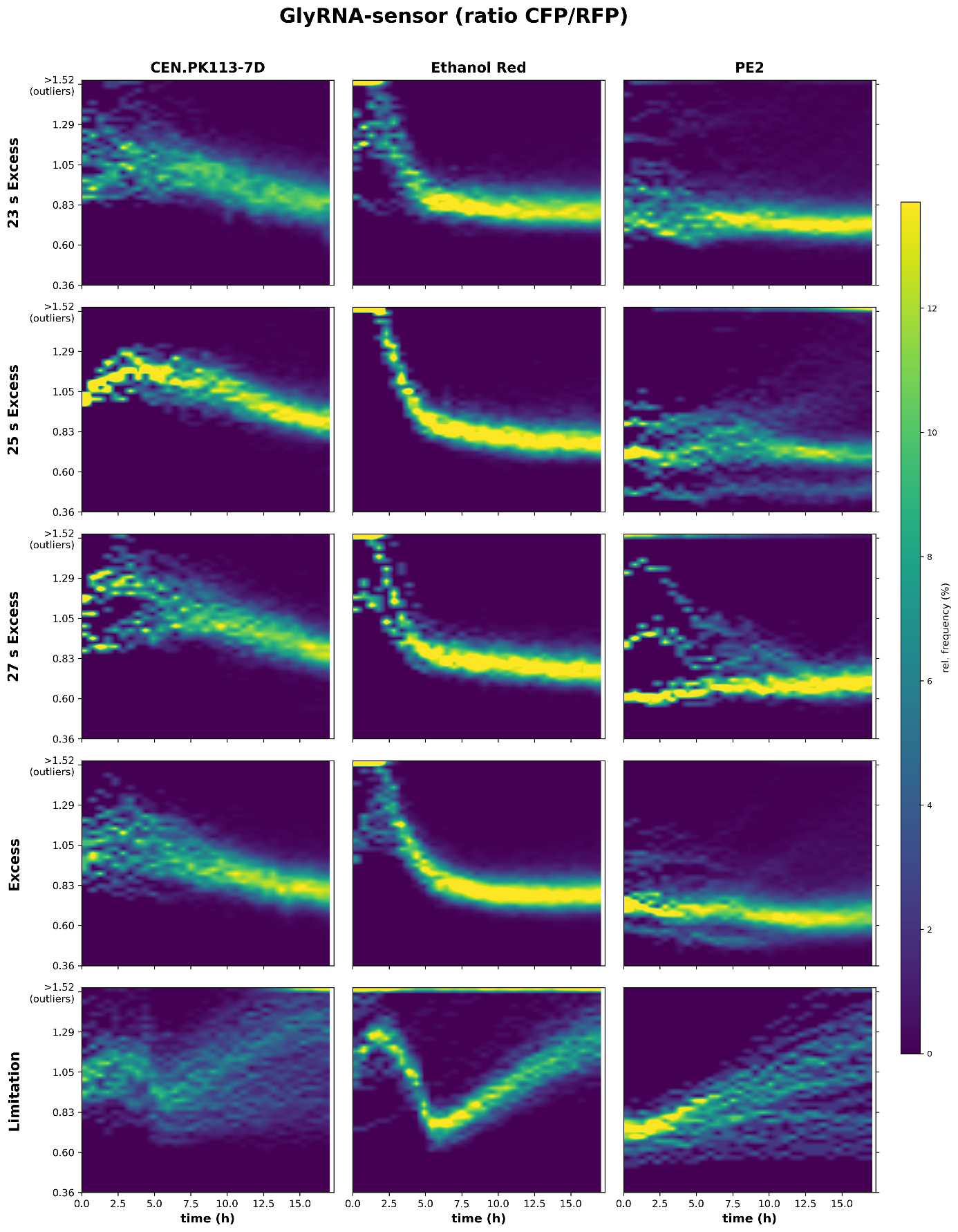


Fig. S13: Heatmaps of the distribution of the GlyRNA sensor (ratio of CFP/RFP) as a proxy for glycolytic flux over time for each tested condition and strain.

One heatmap includes the single-cell data of all recorded FoVs for that strain and condition. The cultivation duration was cropped to the one of the shortest experiments.

## Figure S14


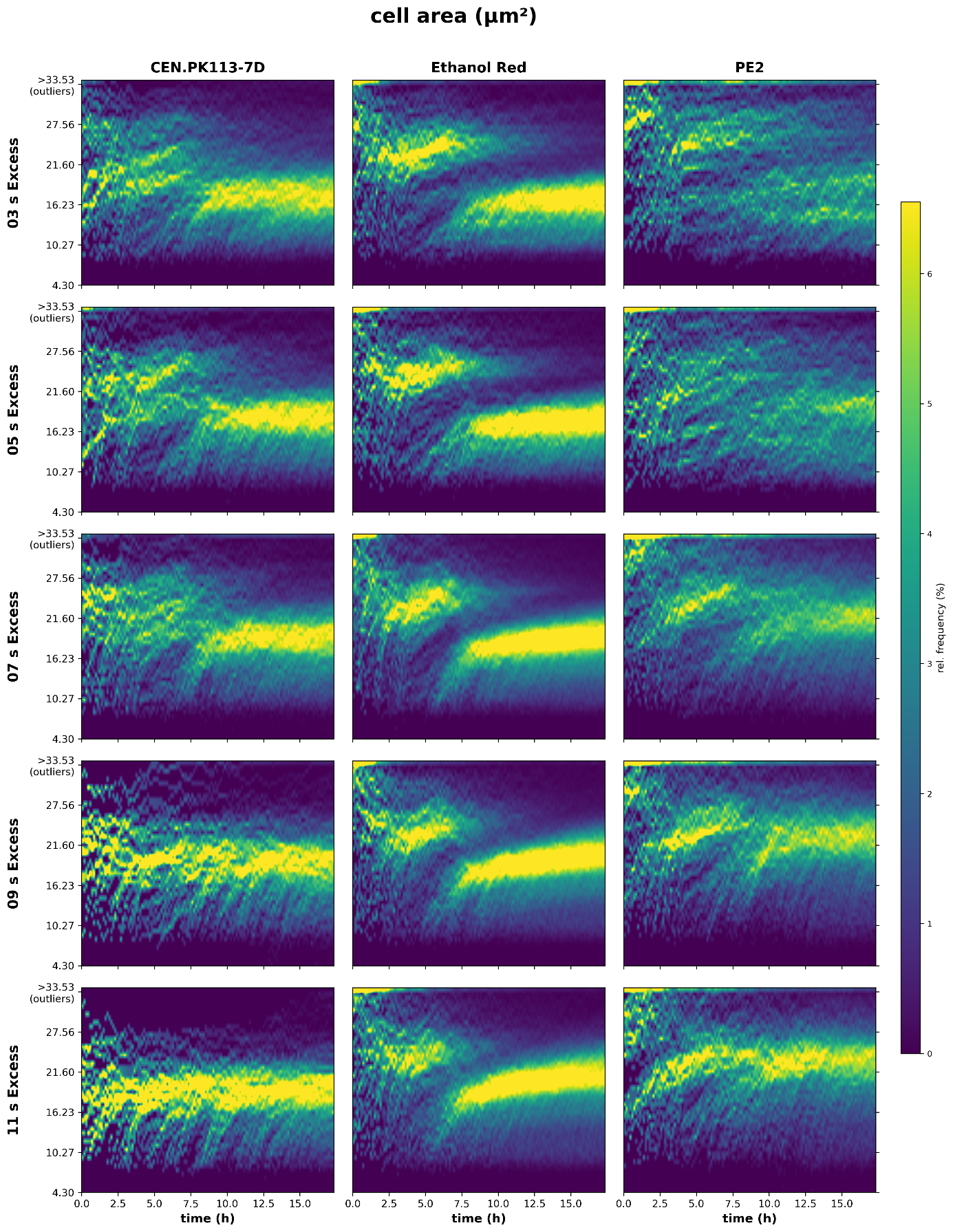


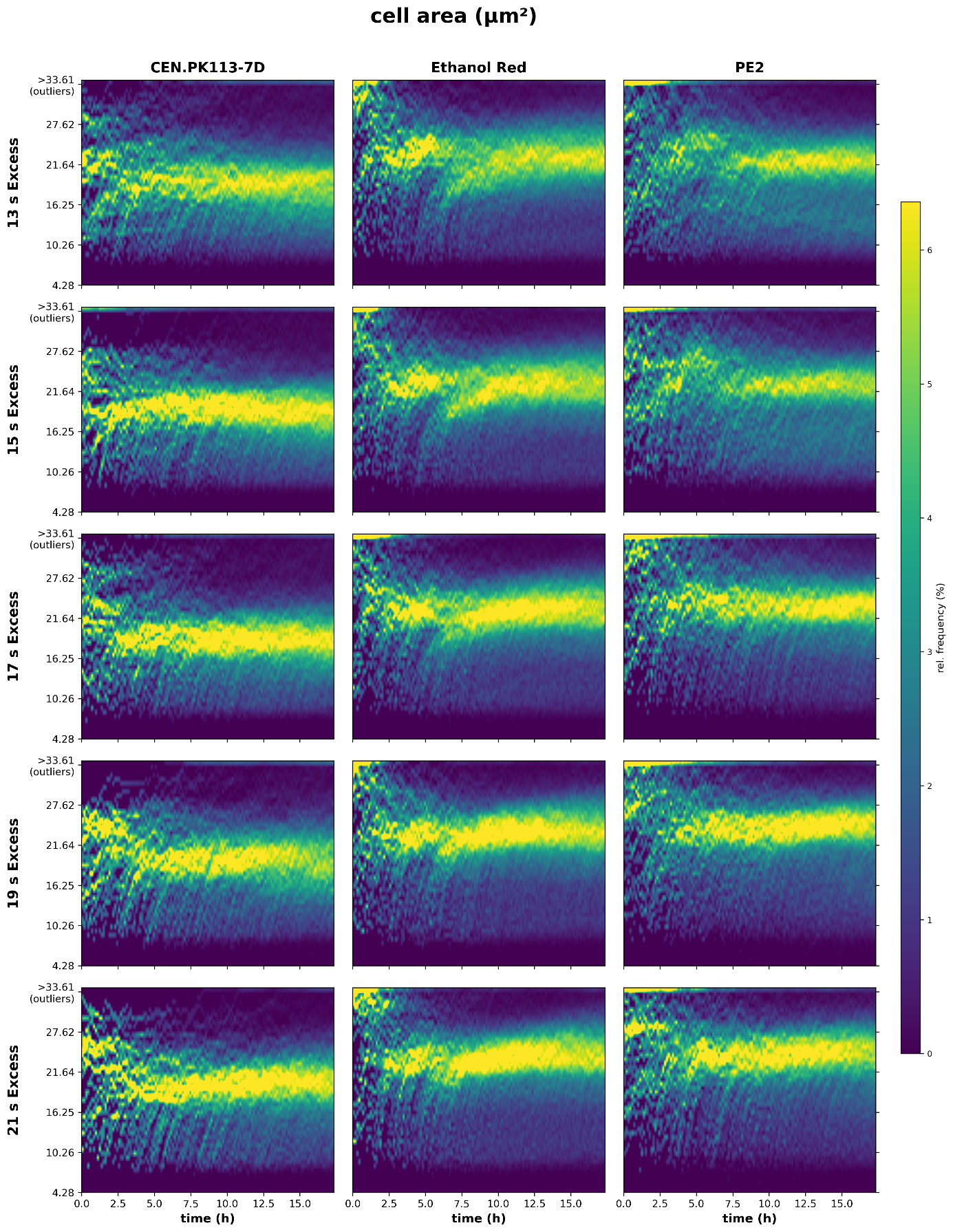


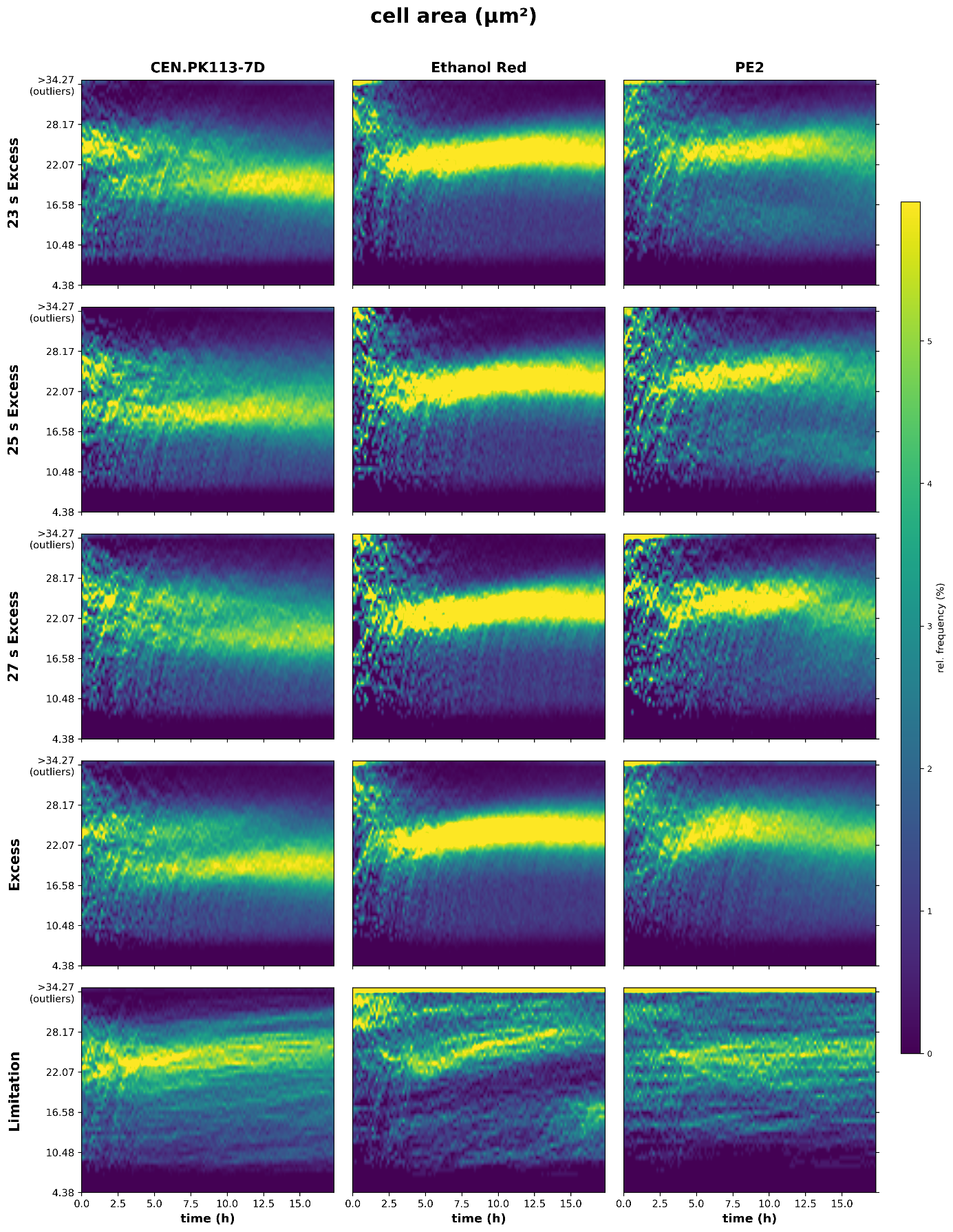


Fig. S14: Heatmaps of the distribution of cell size over time for each tested condition and strain.

One heatmap includes the single-cell data of all recorded FoVs for that strain and condition, combining measurement from QUEEN-2m, GlyRNA and strain with no integrated biosensor. The cultivation duration was cropped to the one of the shortest experiment.

## Figure S15


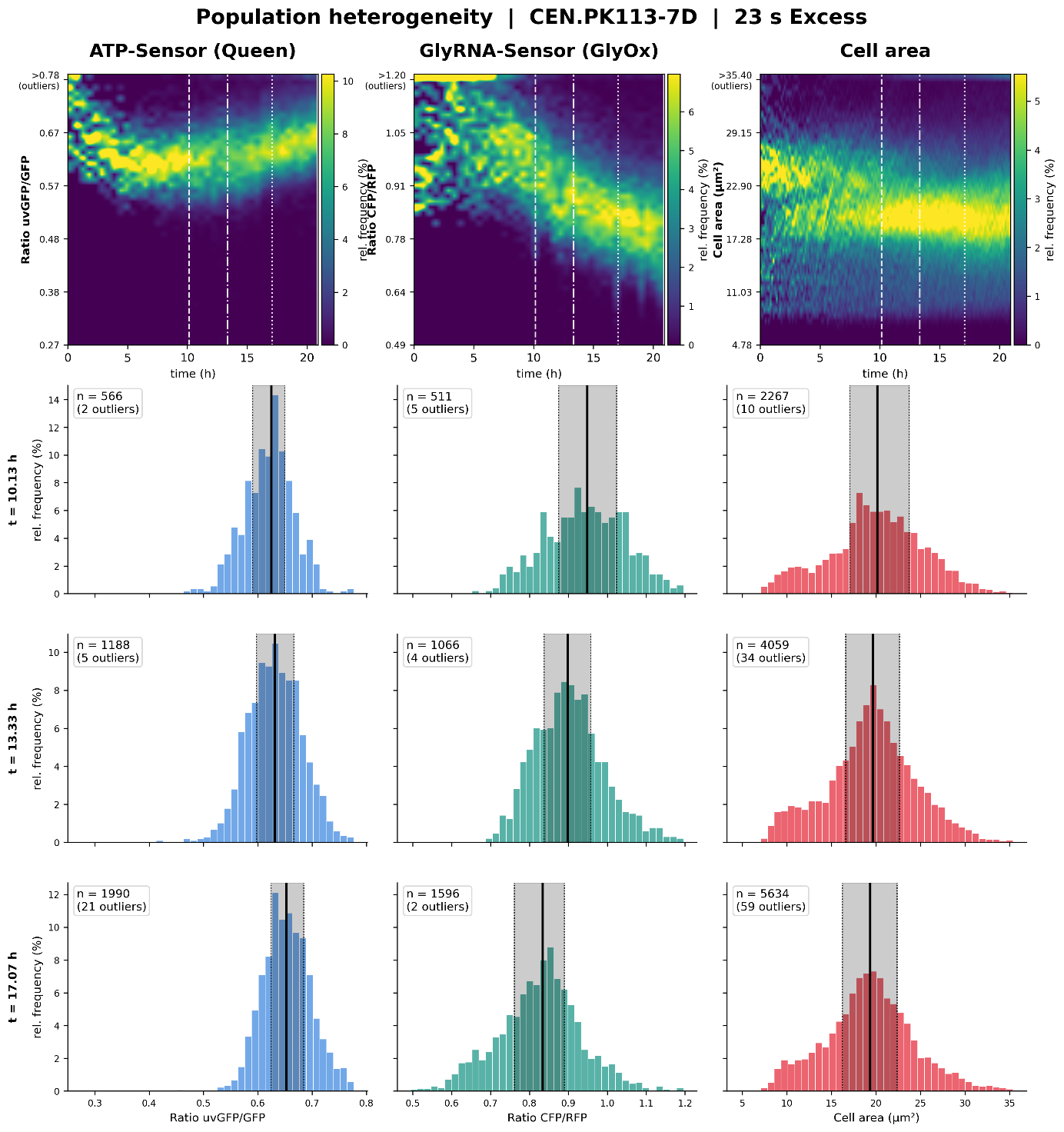


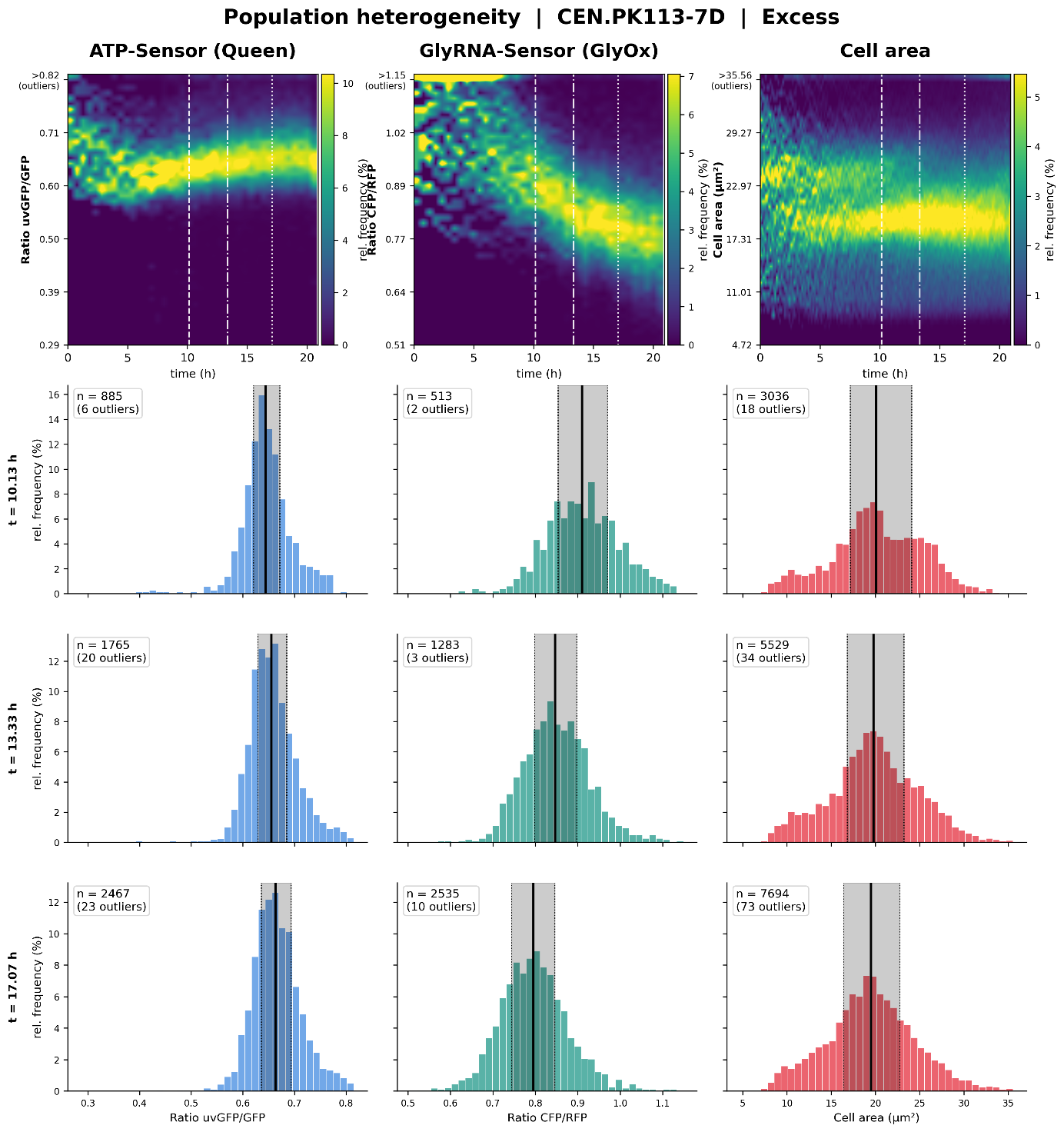


Figure S15: Population heterogeneity of QUEEN-2m (ATP levels), GlyRNA (FBP levels, glycolytic flux) and cell area in CEN.PK113-7D grown in dynamic glucose environments (23 s / 7s oscillation, top) and constant glucose environments (excess control, bottom).

The heatmap show the development of each cellular function over time for CEN.PK113-7D in both dynamic (23 s excess / 7 s limitation) and constant (excess) glucose environments. The histograms focus on the distribution of the cellular function at certain timepoints during the cultivation (see dashed lines in heatmap).

## Figure S16


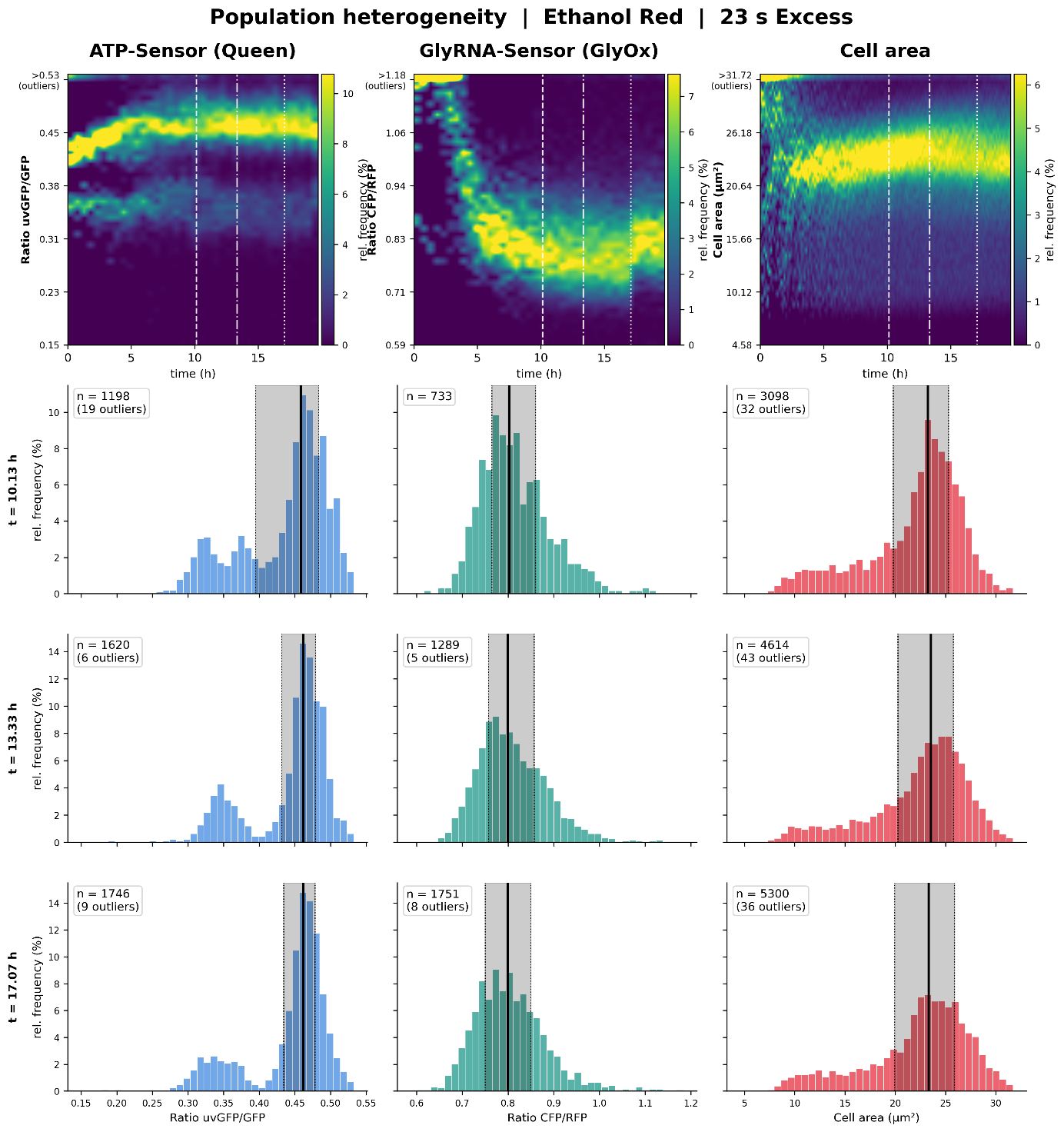


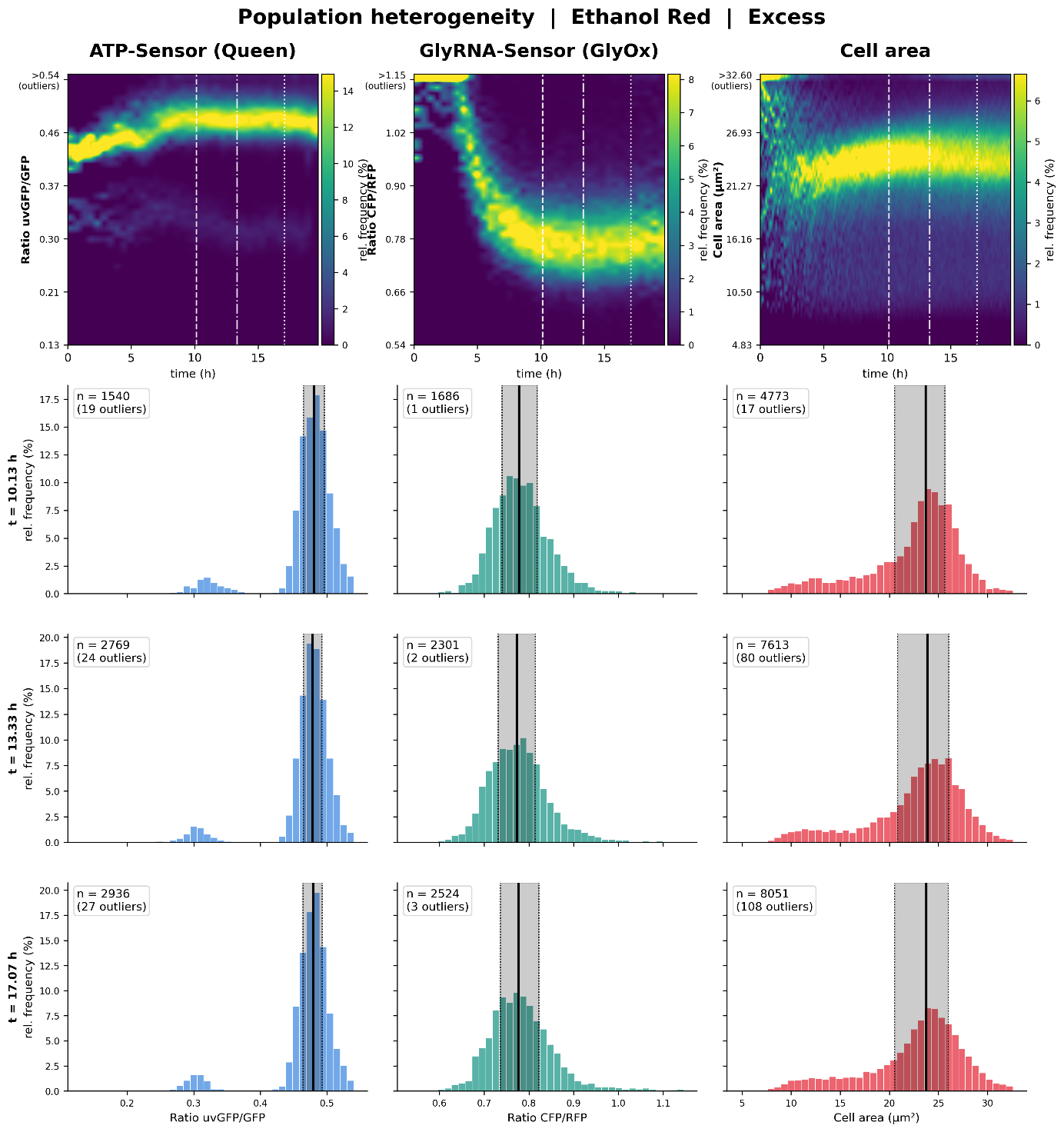


Figure S16: Population heterogeneity of QUEEN-2m (ATP levels), GlyRNA (FBP levels, glycolytic flux) and cell area in Ethanol Red grown in dynamic glucose environments (23 s / 7s oscillation, top) and constant glucose environments (excess control, bottom).

The heatmap show the development of each cellular function over time for Ethanol Red in both dynamic (23 s excess / 7 s limitation) and constant (excess) glucose environments. The histograms focus on the distribution of the cellular function at certain timepoints during the cultivation (see dashed lines in heatmap).

## Figure S17


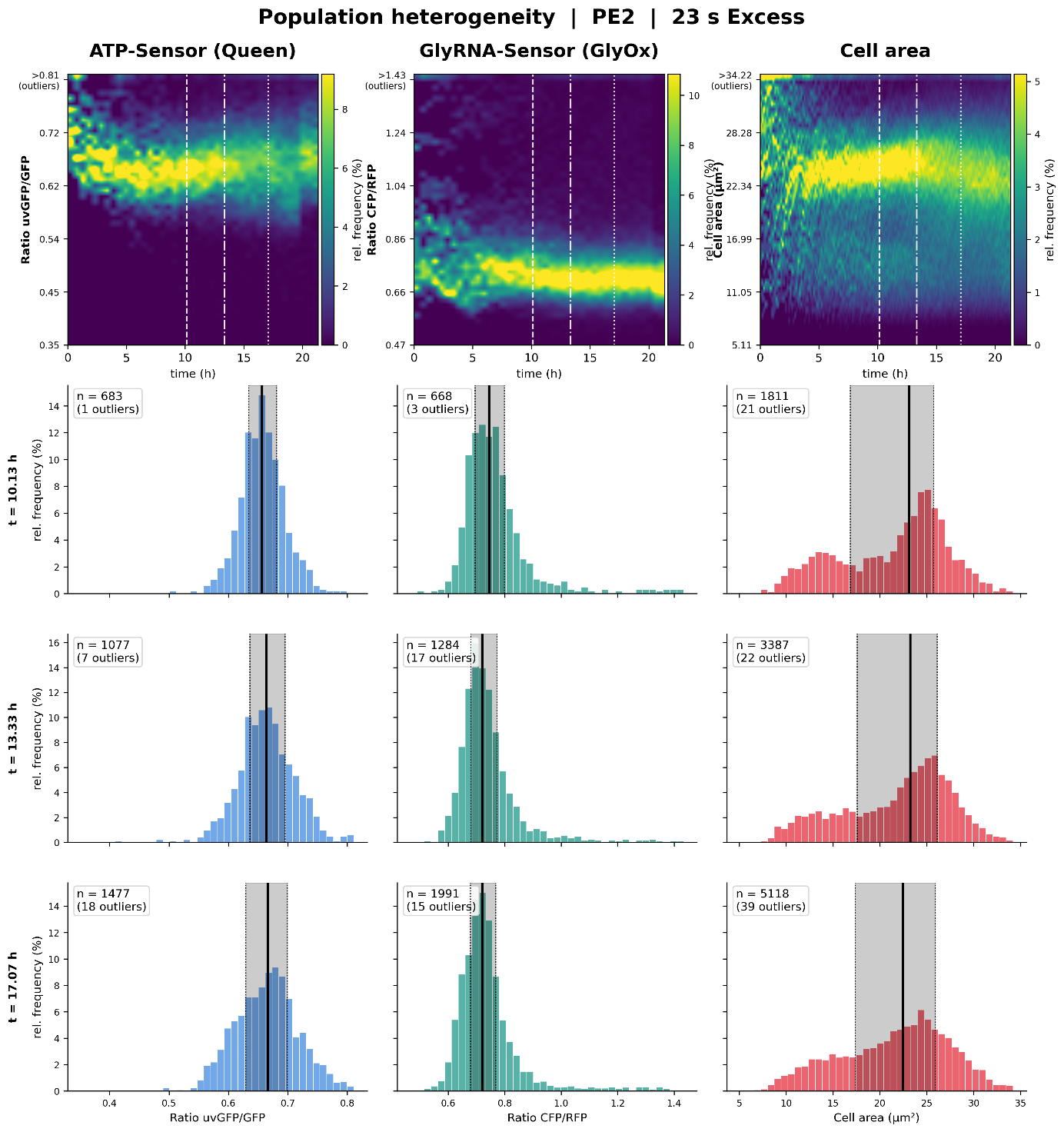


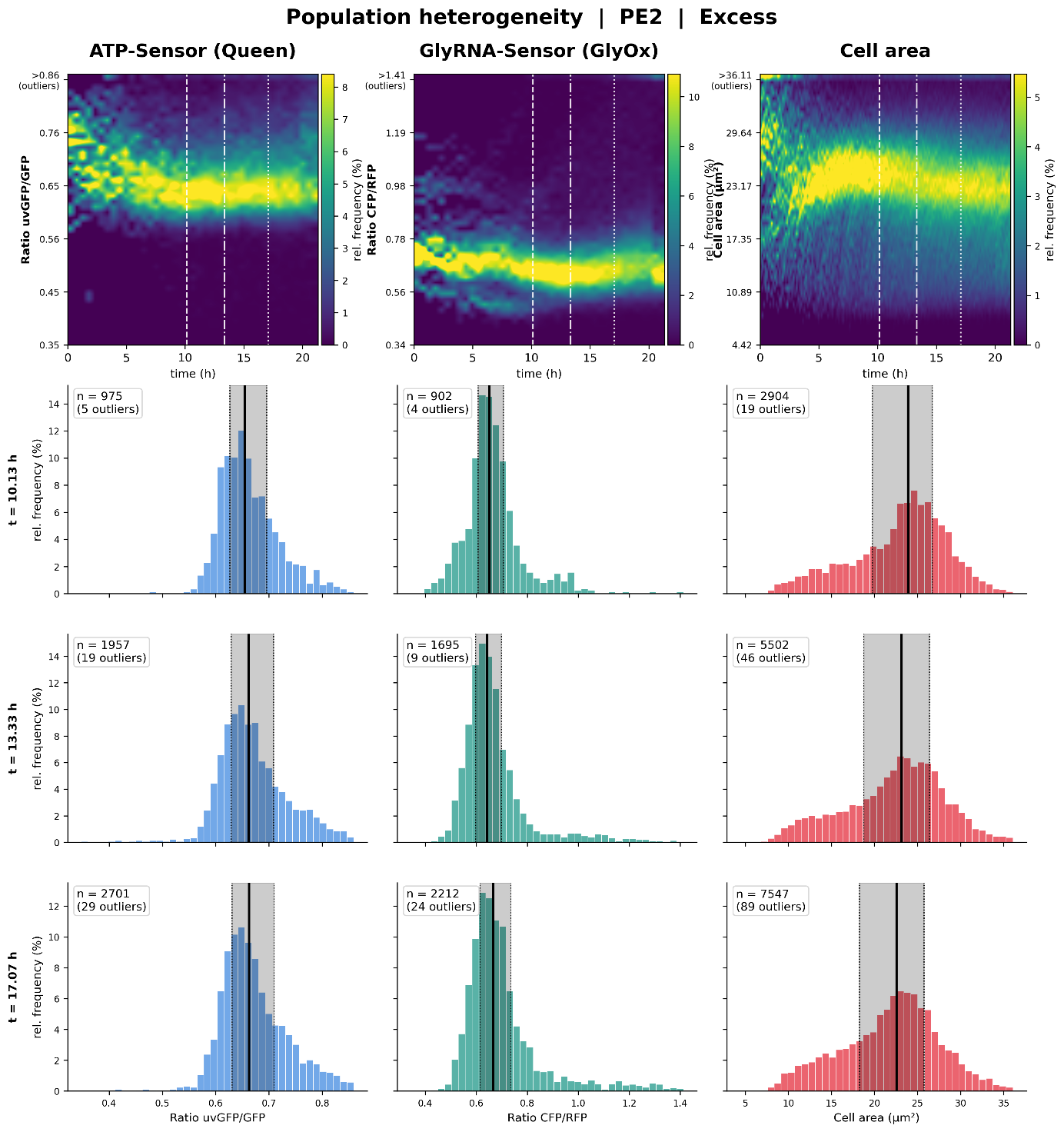


Figure S17: Population heterogeneity of QUEEN-2m (ATP levels), GlyRNA (FBP levels, glycolytic flux) and cell area in PE2 grown in dynamic glucose environments (23 s / 7s oscillation, top) and constant glucose environments (excess control, bottom).

The heatmap show the development of each cellular function over time for PE2 in both dynamic (23 s excess / 7 s limitation) and constant (excess) glucose environments. The histograms focus on the distribution of the cellular function at certain timepoints during the cultivation (see dashed lines in heatmap).
